# *PME10* Is a Pectin Methylesterase Driving PME Activity and Immunity Against *Botrytis cinerea* in Grapevine (*Vitis vinifera* L.)

**DOI:** 10.1111/pbi.70279

**Published:** 2025-07-29

**Authors:** Jorge Lagrèze, Antonio Santiago Pajuelo, Daniele Coculo, Bárbara Rojas, Gaston A. Pizzio, Chen Zhang, Meng‐Bo Tian, Mickael Malnoy, Alessandro Vannozzi, Lorenza Dalla Costa, Vincenzo Lionetti, José Tomás Matus, Giulia Malacarne

**Affiliations:** ^1^ Research and Innovation Center E. Mach Foundation (FEM) San Michele all'Adige (Trento) Italy; ^2^ Center Agriculture Food Environment (C3A) University of Trento/Fondazione Edmund Mach San Michele all'Adige (TN) Italy; ^3^ Institute for Integrative Systems Biology (I2SysBio) Universitat de València‐CSIC Paterna Valencia Spain; ^4^ Department of Biology and Biotechnologies ‘Charles Darwin’ Sapienza University of Rome Rome Italy; ^5^ Center for Viticulture and Enology, College of Food Science and Nutritional Engineering China Agricultural University Beijing China; ^6^ Department of Agronomy, Food, Natural Resources, Animals and Environment (DAFNAE) University of Padova Legnaro (PD) Italy

**Keywords:** cell wall integrity, CRISPR/Cas9, expression atlas, gene co‐expression network, gene overexpression, plant immunity, PME, RNA‐seq

## Abstract

*Botrytis cinerea* (Bc) is a major pathogen of cultivated grapevine ( *Vitis vinifera* L.), with cell wall (CW) remodelling playing a critical role in fungal colonisation. CW‐modifying enzymes, particularly pectin methylesterases (PMEs), produced by both host and pathogen, influence CW integrity and the outcome of infection. To explore the role of CW composition and remodelling in grapevine's response to Bc, we inoculated three genotypes with varying susceptibility at full flowering. Biochemical analysis of flowers and ripe berry skins revealed that the tolerant genotype exhibited significantly higher PME activity postinfection compared with the susceptible ones. Unbiased transcriptome analysis of infected flower tissues showed a more intense transcriptional response in the susceptible genotype, suggesting an ultimately ineffective attempt to restrict fungus spread. Expression profiling of 62 *PME* genes in this data set and public Bc‐infected berry transcriptomes identified *PME10* as the most strongly induced gene upon infection. *PME10* knockout mutants displayed reduced PME activity and heightened susceptibility, while overexpression lines showed enhanced PME activity and reduced disease symptoms. Gene co‐expression network analysis highlighted WRKY03, a defence‐related transcription factor, as a putative regulator of *PME10*. DAP‐seq, DAP‐qPCR and dual luciferase assays confirmed direct binding and activation of the *PME10* promoter by WRKY03. Altogether, this study demonstrates that PME10 is a functional PME contributing to grapevine immunity against *B. cinerea* , establishing it as a key component of the grapevine defence machinery against fungal pathogens.

## 1 Introduction

The necrotrophic fungus *Botrytis cinerea* (Bc) is one of the most important pathogens affecting cultivated grapevine ( *Vitis vinifera* L.) particularly under favourable environmental conditions (Rahman et al. 2018). Bc causes grey mould, also known as Botrytis bunch rot, which develops primarily on ripe berries, leading to substantial reductions in both fruit yield and quality of wine and table grapes at harvest (Kelly et al. 2022).

During the early stages of infection, Bc forms specialised structures called appressoria, which facilitate host colonisation through secretion of effectors capable of degrading the cuticle and cell wall (CW) (Choquer et al. 2021). As fungal cell wall‐modifying enzymes (CWMEs) are key virulence factors for Bc pathogenesis (Blanco‐Ulate et al. 2014), it has been proposed that CW degradation contributes significantly to plant susceptibility (Cantu et al. 2008). Conversely, the plant CW serves not only as a physical barrier but also as a dynamic component of the immune response, playing a critical role in restricting pathogen invasion (Bellincampi et al. 2014; Malinovsky et al. 2014).

Berry ripening is often accompanied by a marked increase in susceptibility to fungal pathogens, particularly *B. cinerea* (Weiller et al. 2021). This heightened vulnerability is associated with berry softening, a process driven by cell wall (CW) remodelling through the activity of specific CWMEs (Malacarne et al. 2024). During its interaction with the berry, Bc secretes a range of CWMEs, including several pectin‐modifying enzymes, which target and degrade the pectic network to facilitate host tissue colonisation (Li et al. 2022). Homogalacturonan (HG), a linear polymer of galacturonic acid, is a primary target of pathogen‐derived pectin‐degrading enzymes. HG is synthesised in the Golgi apparatus and delivered to the cell wall in a highly methylesterified form (Harholt et al. 2010; Ibar and Orellana 2007). Its degree of methylesterification is regulated *in muro* by pectin methylesterases (PMEs), which hydrolyse the methyl ester bonds at the C‐6 position of galacturonic acid residues in the apoplast, generating de‐esterified (acidic) pectins while releasing methanol (MeOH) and protons (Körner et al. 2009). Notably, PME activity is strongly induced upon pathogen attack and plays a key role in plant defence responses against *B. cinerea* and other pathogens (Bethke et al. 2014; Lionetti et al. 2012; Raiola et al. 2011). Among the various isoforms involved in these responses, *Arabidopsis* AtPME17 is widely induced during infection by multiple pathogens and is considered a general biomarker of pathogenesis (Del Corpo et al. 2020).

Plant PMEs play diverse roles in modulating plant immunity (Del Corpo et al. 2024). By generating negatively charged regions in homogalacturonan (HG), PMEs facilitate calcium‐mediated crosslinking, which reinforces the cell wall and limits pathogen ingress (Coculo et al. 2023). PME activity, in coordination with polygalacturonases (PGs) and their inhibitors (PGIPs), also promotes the release of demethylesterified oligogalacturonides (OGs), which function as damage‐associated molecular patterns (DAMPs) to trigger immune responses (Osorio et al. 2011). In addition, PMEs enhance immune signalling by increasing the affinity of surface receptors for demethylesterified pectin (Lin et al. 2022). The interaction between RALF peptides and OGs further amplifies immune activation (Liu et al. 2024). Lastly, the demethylesterification of pectin is the main source of plant‐derived methanol (MeOH), which acts as a volatile alarm signal during pathogen attack (Hann et al. 2014).

Genome‐wide identification and characterisation of different PME isoforms, whether constitutively expressed or developmentally regulated, have been performed in diverse plant species, including Arabidopsis (Louvet et al. 2006), rice (Jeong et al. 2015), maize (Zhang et al. 2019), tomato (Wen et al. 2020), strawberry (Xue et al. 2020), soybean (Wang et al. 2021) and grapevine (Khan et al. 2019). Specific PME isoforms have been implicated in modulating fruit susceptibility to Bc (Cantu et al. 2009; López‐Casado et al. 2023; Ortega‐Salazar et al. 2024; Osorio et al. 2011; Silva et al. 2023).

In grapevine, several studies have demonstrated a correlation between cell wall (CW) composition, particularly the degree of homogalacturonan (HG) methylesterification, and cultivar‐specific susceptibility to *B. cinerea* (André et al. 2021; Weiller et al. 2021). Infection‐induced changes in CW composition are often accompanied by transcriptional reprogramming of CWME‐encoding genes, with responses varying across cultivars and developmental stages (Agudelo‐Romero et al. 2015; Haile et al. 2017, 2020; Kelloniemi et al. 2015). These transcriptional shifts are further modulated by the type of rot—bunch rot or noble rot—that develops during infection (Amrine et al. 2015; Hegyi et al. 2022; Lovato et al. 2019; Váczy et al. 2024). Focusing on the grape (*Vvi*) PME family, specific members are induced in the ripe berries of ‘Pinot Noir’ and cv. ‘Semillon’ cultivars during Bc infection (Malacarne et al. 2024).

In this study, we investigated the role of pectin modification, mediated by PME activity, in the grape response to Bc. Among the 62 annotated *PME* genes in grapevine, the *PME10* gene emerged as a key candidate due to its strong induction upon Bc infection and its high expression in overripe berries, a developmental stage particularly susceptible to the pathogen. Functional characterisation using gene knockout and overexpression approaches demonstrated that *PME10* contributes to resistance against Bc. These findings identify *PME10* as a novel genetic determinant of grapevine defence and provide a promising target for the development of Bc‐resistant cultivars using advanced New Genomic Techniques.

## 2 Results

### 2.1 *Botrytis cinerea* Infection Caused Higher Colonisation in ‘Teroldego’ and Blossom Abortion in ‘Sangiovese’ Compared to 'Souvignier Gris'

*Botrytis cinerea* is a major pathogen in viticulture, causing substantial damage to both flowers and berries. To investigate its impact on grapevines, we selected three genotypes with contrasting susceptibility: The interspecific hybrid ‘Souvigner Gris’ (SG), which is resistant to the fungus, and the *V. vinifera* cultivars ‘Sangiovese’ (SN) and ‘Teroldego rotaliano’ (TE), which are both reported to be susceptible (Rahman et al. 2018; Vezzulli et al. 2022). SG is a white wine variety with robust berry skins that reduce the risk of Bc infection, even in areas with high rainfall (Casanova‐Gascón et al. 2019). TE, which is cultivated in the Rotaliana plain and is of local significance but is highly susceptible to the fungus in wet years. Meanwhile, SN is the most widely planted Italian variety. It is valued for its versatility in wine production, but it is also highly susceptible to Bc (Galet 2000).

**FIGURE 1:**
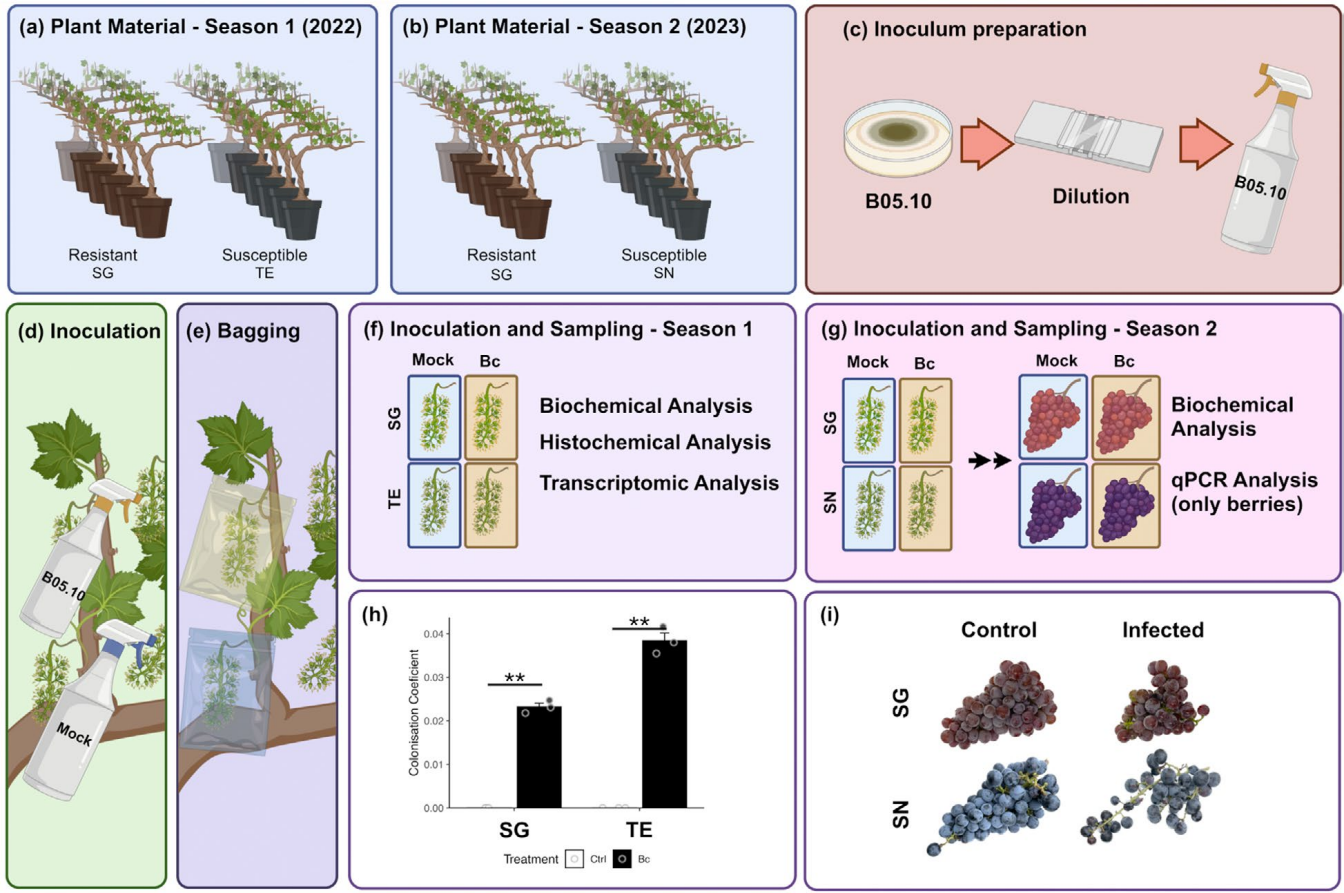
*Botrytis cinerea* inoculation assays of grapevine genotypes with contrasting susceptibility. Three grapevine genotypes with varying susceptibility to the fungus were selected: ‘Souvignier Gris’ (SG), resistant and the *V. vinifera* cultivars. ‘Teroldego rotaliano’ (TE) and ‘Sangiovese’ (SN), both susceptible. Two independent inoculation experiments were conducted at full flowering in consecutive seasons. (a) In Season 1 (2022), SG and TE were compared to investigating early stages of infection in flowers. (b) In Season 2 (2023), SG and SN were compared to assessing both early (flowers) and late (berries) infection stages. (c–e) In both experiments, inflorescences at full cap‐fall stage (E‐L 25/26) were inoculated with a *Bc* suspension (2 × 10^5^ conidia/mL) or mock‐inoculated with conidia‐free medium. Samples were enclosed in clear plastic bags and sprayed with 50 mL of water to maintain high humidity for 24 h, promoting conidial germination. (f) In season 1, flower samples were collected at 24 h post‐inoculation (hpi) and analysed at the biochemical, histochemical and transcriptional levels (RNA‐seq). (g) In season 2, flowers and berry samples were collected at 24 and 96 hpi, and at 12 weeks post‐inoculation (wpi), respectively, and analysed at biochemical and transcriptional (qPCR) level. All samples were stored at −80°C until analysis. The artwork was created with BioRender.com. (h) Coefficient of colonisation in inoculated and control inflorescences of SG and TE, calculated as the ratio of *B. cinerea* to *V. vinifera* DNA, quantified by qPCR bars represent the mean, and whiskers indicate the standard error of the mean (SEM). Statistical significance was assessed using Student's *t*‐test (**p‐value ≤ 0.01). (i) Representative images of grape clusters from SG and SN genotypes taken at 12 wpi, either inoculated with *B. cinerea* (infected) or mock‐inoculated (control).

Two independent inoculation experiments were conducted at full flowering (EL 25–26) over two consecutive seasons (2022 and 2023). In the first season, SG and TE were compared to investigate the early infection stages. Therefore, all inflorescences from both genotypes were collected 24 h post‐inoculation (hpi) with Bc or with a mock treatment (control medium without conidia) and analysed biochemically, histochemically and transcriptionally via RNA‐seq (Figure 1a,c–f). In the second season, the same replicates of SG were compared with SN to validate previous findings and to assess later stages of infection. Half of the inoculated inflorescences were collected at 24 and 96 hpi, while the rest were retained until berry ripening and collected at 12 weeks post‐inoculation (wpi) for biochemical and qPCR‐based transcriptional analysis (Figure 1b–e,g).

The presence of Bc in flower samples was confirmed in both SG and TE by PCR amplification of the Bc3 marker in genomic DNA from inoculated tissues, validating the inoculation procedure. However, at 24 hpi, the colonisation coefficient was significantly higher in TE, indicating greater fungal proliferation (Figure 1h). At 12 wpi, SN bunches did not show typical grey mould symptoms but exhibited signs of poor fruit set, indicative of flower abortion likely triggered by infection at flowering (Figure 1i). These findings confirmed that TE and SN are considerably more susceptible to Bc than SG.

### 2.2 Higher Grapevine Resistance to *B. cinerea* Positively Correlates With Increased PME Activity

To understand the potential role of the cell wall (CW) in the grapevine's response to the fungus, we tested whether the different levels of resistance to Bc observed in different tissues could be related to CW composition and/or remodelling. Flowers (F) and berry skins (BS) were analysed for CW monosaccharide composition, revealing few differences in their composition. Galacturonic acid was the main monosaccharide both in flowers (F) (40%) and berry skins (BS) (50%), followed by galactose (19%) in F and arabinose (19%) in BS. A lower content of glucose (10%‐F and 10%‐BS) and xylose (8.5%‐F and 7%‐BS) and a small amount of rhamnose (3% in both cases), mannose (2.5%‐F and 2%‐BS), fucose (1.5% in both cases) and glucuronic acid (0.5% in both cases) were revealed in both tissues (Figure S1). However, monosaccharide composition was similar between cultivars, treatments and time after treatment. The CW composition of the resistant SG was similar to that of the susceptible TE and SN. CW composition was not affected by Bc infection in the three cultivars at 24, 96 and 12 wpi, suggesting that the difference in response to Bc of the different genotypes is not related to CW polysaccharide content.

**FIGURE 2:**
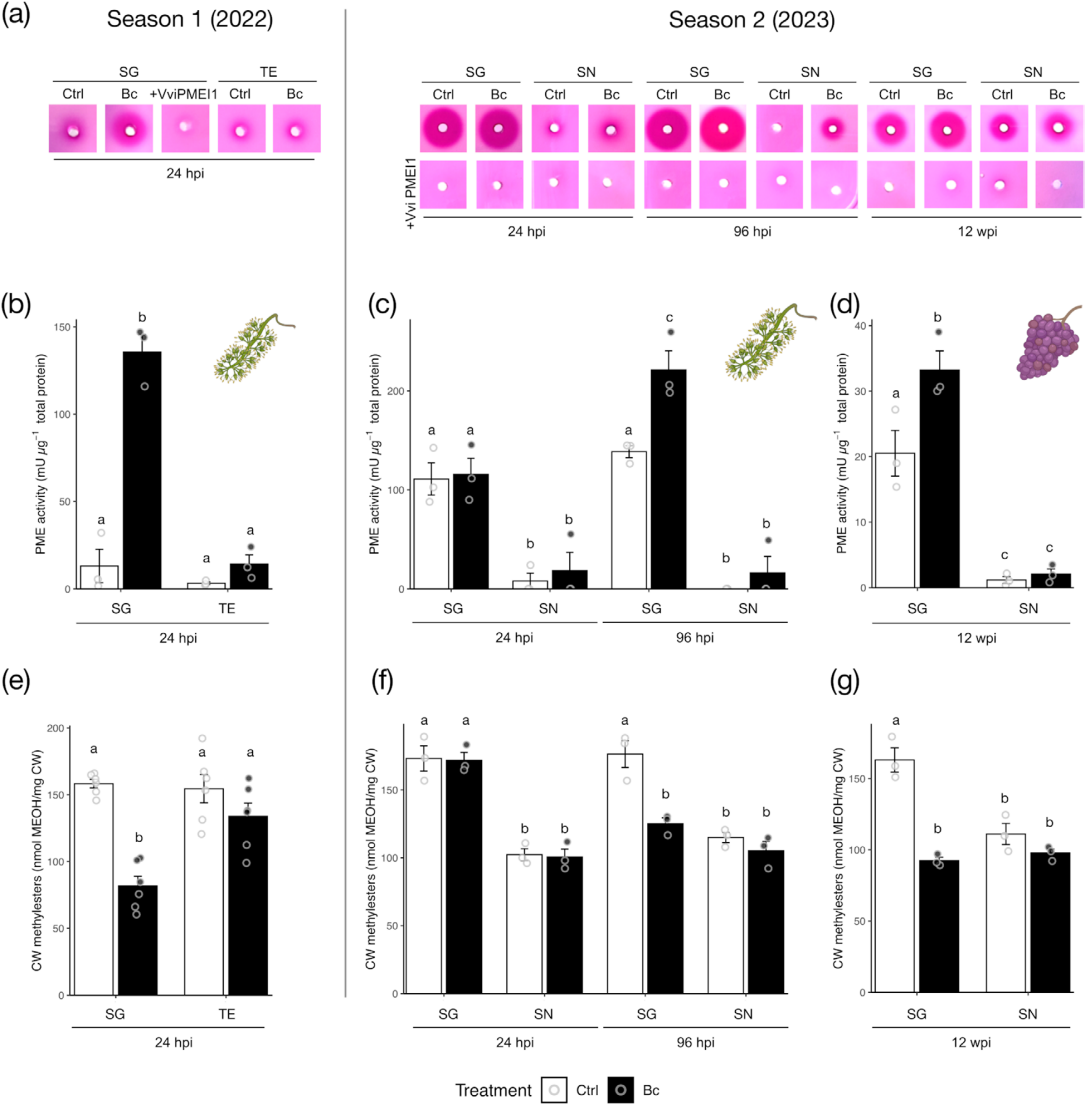
Higher grapevine resistance to *B. cinerea* positively correlates with increased PME activity. (a–d) Quantification of PME activity in protein extracts from: (b) uninfected and infected flowers of ‘Souvignier Gris’ (SG) and ‘Teroldego’ (TE), collected at 24 h post‐inoculation (hpi) during Season 1; (c) uninfected and infected flowers of SG and ‘Sangiovese’ (SN) collected at 24 and 96 hpi during Season 2; (d) uninfected and infected berry skins of cv. ‘Souvigner Gris’ and cv. ‘Sangiovese’ collected at 12 wpi (Season 2). Data are presented as mean ± SD (*n* = 3). In Panel (a), the results of the PECTOPLATE assay are visualised, showing the PME activity (fuchsia halo) in total protein extracts from all samples analysed. +VviPMEI1 = PME inhibitor from *V. vinifera* exogenously added to protein extracts. (e–g) Quantification of methyl ester content in the same samples analysed for PME activity. Results represent mean ± SD (*n* = 6). Different letters on the bars indicate statistically significant differences based on ANOVA followed by Tukey's test (*p* < 0.05). Bc, *B. cinerea* ; Ctrl, Control.

The potential involvement of grapevine‐derived PME activity in the response to *B. cinerea* was subsequently investigated. The previously described flower and berry samples were analysed for PME enzymatic activity (Figure 2). No significant differences in PME activity were observed between flowers of mock‐inoculated resistant genotype SG and susceptible TE in season 1 (Figure 2b). However, in season 2, SG exhibited significantly higher basal PME activity compared with the susceptible SN in both flowers (Figure 2c) and berry skins (Figure 2d). Upon infection, PME activity in SG showed a notable induction, already at 24 h post‐inoculation (hpi) in flowers during Season 1, and from 96 hpi in season 2 (Figure 2b,c). A similar pattern was observed in berry skins (Figure 2d). In contrast, no significant change in PME activity was detected in infected TE (Figure 2b) or SN (Figure 2c,d) tissues compared to their respective controls. Thus, in both seasons, the resistant SG genotype showed a marked induction of PME activity upon infection, though with a temporal delay in season 2.

Given that *B. cinerea* produces its own PME to facilitate infection, it was necessary to determine whether the PME activity observed in infected tissues of SG originated from the host or the pathogen. To this end, PME inhibition assays were performed using VviPMEI1, a functional *V. vinifera* PME inhibitor known to be ineffective against fungal PMEs (Lionetti et al. 2015). The addition of VviPMEI1 to infected SG tissue extracts completely abolished PME activity (Figure 2a), confirming that the induced activity was of grapevine origin. These results support a role for host‐derived PME activity in the grapevine's defence response to *B. cinerea* .

### 2.3 Higher Resistance to *B. cinerea* Is Associated With a Lower Level of Unesterified Pectin

**FIGURE 3:**
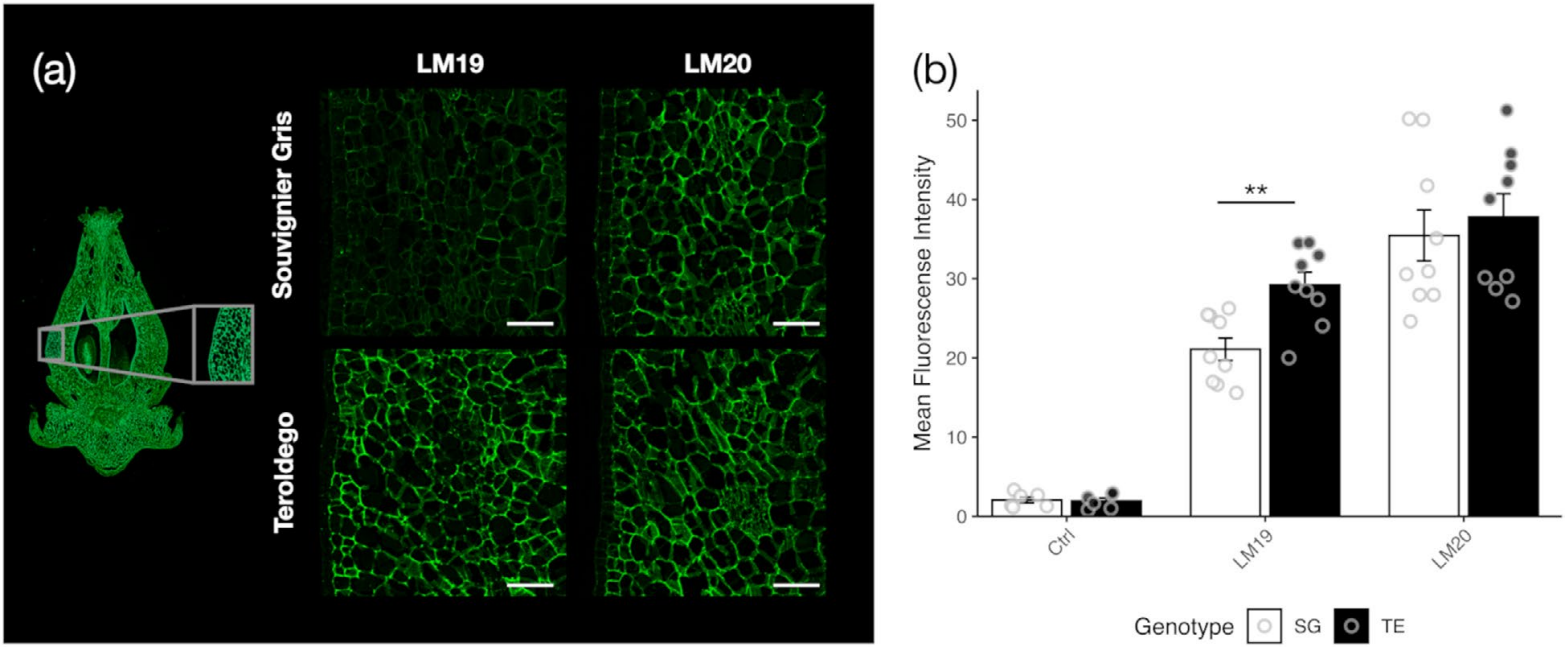
Increased resistance to *B. cinerea* is associated with lower levels of unesterified pectin. (a) Left: Representative image of a whole flower indicating the region analysed. Right: Immunolabelling of demethyl esterified pectin (LM19) and methyl esterified pectin (LM20) in inflorescences of Souvignier Gris (SG) and ‘Teroldego’ (TE). Both LM19 and LM20 primary antibodies were detected using AlexaFluor 488 Plus‐conjugated secondary antibodies. Scale bar = 50 μM. (b) Quantification of mean Fluorescent Intensity in ‘SG’ and ‘TE’ samples for LM19, LM20, and their respective controls (samples processed without primary antibody), performed using LASX software (Leica). Statistically significant differences were assessed using Student's *t*‐test (**, adjusted *p*‐value ≤ 0.01).

To investigate whether differences in resistance to Bc are associated with different levels of methyl/demethylesterified pectin, the CW methylester content was quantified in all tissues analysed. Compared with the control, a significant reduction in methylester content was observed in the resistant cultivar upon Bc infection, which correlated with the significantly higher PME activity detected in both flower and berry skin compared with the control. This reduction was not observed in the susceptible genotypes (Figure 2e–g). Next, the methylesterification status of pectin was compared in flower tissues of SG and TE by immunohistochemistry, using LM19 and LM20 antibodies, which preferentially recognise demethylesterified and methylesterified pectin, respectively. No significant difference in LM20‐labelling distribution and intensity was detected between SG and TE flower tissues. Instead, a lower fluorescence signal was observed in LM19 labelling in SG with respect to TE (Figure 3). This result reveals a lower level of unesterified pectin epitope in SG flowers with respect to TE.

### 2.4 Genome‐Wide Identification and Phylogenetic Characterisation of the VviPME Gene Family

A total of 62 members of the *Pectin Methyl Esterase* (*PME*) gene family were identified considering two different *V. vinifera* PN40024 reference genome assemblies (12X.v2 and PN40024.v4.40X) and annotations (VCost.v3 and V4) (Canaguier et al. 2017; Velt et al. 2023; Table S1). Of the 62 genes, 61 were identified in both genome annotations, while one (Vitvi07g04792) was exclusively detected in the V4 annotation of the PN40024.v4.40X assembly. In addition to the 47 PMEs identified in a previous study by Khan et al. (2019), 15 new PME genes were identified. Of these, 47 were named according to Khan et al. (2019), while the remaining 15 were named according to the phylogenetic relationships discovered in the present study. The structure of the identified genes was manually curated according to Velt et al. (2023). Of the 62 genes, 39 were manually curated using publicly available *V. vinifera* transcriptomic data, and 23 could not be confirmed due to a lack of transcriptomic data mapping to the respective genes. Based on the PME classification proposed by (Pelloux et al. 2007), 30 of the 62 gene members were classified as Group I PMEs, characterised by the presence of the PME domain (Pfam01095) only; the other 32 were classified as Group II PMEs, characterised by both the PME domain (Pfam01095) and the PRO region (Pfam04043).

A phylogenetic analysis of the *PME* gene families in *V. vinifera* and *A. thaliana* grouped the identified isoforms into 10 major clades, along with two orphan groups (Figure S2). Notably, six grapevine *PME* genes (*VviPME8*, *VviPME9*, *VviPME10*, *VviPME11*, *VviPME12*, *VviPME54*) clustered within clade 5A, which also includes *AtPME17*, a known contributor to *Arabidopsis* resistance against *B. cinerea* (Del Corpo et al. 2020). Gene expression profiling across organs and developmental stages, using the cv. ‘Corvina’ Atlas Explorer (http://plantaeviz.tomsbiolab.com/vitviz/corvina_atlas/) revealed organ‐ and stage‐specific patterns for *PME* gene expression (Figure S3). Members of clade 5A exhibited their highest expression in berry tissues during postharvest withering (PHW), indicative of a role in fruit over‐ripening. An exception was *VviPME12*, which showed maximal expression in roots and pollen. Although clade 5A genes were generally lowly expressed in flowers, their expression increased progressively during floral development. In addition, other *PME* genes, *VviPME13*, *VviPME17*, *VviPME19*, *VviPME32* and *VviPME39*, were also highly expressed during PHW. Conversely, a distinct subset of *PME* genes, including *VviPME14, VviPME16, VviPME24, VviPME27, VviPME28, VviPME36, VviPME43* and *VviPME56*, displayed strong and specific expression during flower development, particularly in stamens and pollen. These observations highlight a finely tuned spatio‐temporal regulation of *VviPME* expression and suggest diverse functional roles across grapevine tissues and developmental stages. The full list of *VviPME* genes, including gene identifiers from the three PN40024 genome annotations, clade classifications and phylogenetic assignments, is provided in Table S1.

### 2.5 A Stronger Transcriptional Response Was Observed in the Susceptible Genotype at Early Stages Post‐ *B. cinerea* Infection

To assess the early transcriptional response to Bc infection and its effect on PME gene expression, RNA‐seq analysis was performed on flowers from ‘Souvignier Gris’ (SG, resistant) and ‘Teroldego’ (TE, susceptible) collected at 24 h post‐inoculation (hpi). The analysis revealed a markedly stronger transcriptional activation in TE compared to SG (Table S3). Specifically, 1738 genes were exclusively upregulated in TE, in contrast to only 103 in SG. Similarly, 1405 genes were exclusively downregulated in TE, whereas SG showed 616 downregulated genes (Figure S4a). Among the 654 genes commonly upregulated in both genotypes, expression levels were significantly higher in TE, particularly for stress‐related genes (highlighted in red in the regression analysis plot, Figure S4b). This suggests an intense, yet ultimately ineffective, defence attempt in the susceptible genotype. Conversely, SG appears to mount a more restrained transcriptional response, likely due to constitutive or pre‐primed resistance mechanisms that require fewer transcriptional changes to restrict pathogen progression. Gene set enrichment analysis supported this interpretation, revealing over‐represented functional categories related to defence and stress responses in both genotypes, though with differing intensity (Figure S4c). Notably, TE exhibited strong upregulation of genes encoding pathogenesis‐related (PR) proteins and transcription factors previously implicated in resistance pathways (Figure S4d). These included *PR10.1* and *PR10.3*, which are among the most responsive *PR* genes in flowers and berries of ‘Pinot Noir’ upon Bc infection (Haile et al. 2017, 2020), as well as MYB14, MYB15 and WRKY03, key regulators of stilbene biosynthesis and associated with resistance mechanisms (Orduña et al. 2022).

### 2.6 Clade 5A PMEs Are Significantly Induced Upon *B. cinerea* Infection in Flowers and Berries

To explore conserved expression patterns of the grapevine *PME* gene family during Bc infection, a publicly accessible visualisation tool, the Botrytis Stress Atlas, was developed and integrated into the Vitis Visualisation (VitViz) platform (http://plantaeviz.tomsbiolab.com/vitviz/botrytis_atlas/). This tool also enabled the retrieval and comparative analysis of *PME* gene expression profiles across multiple data sets of Bc‐infected grapevine flowers and berries. The raw data from the flower RNA‐seq experiment, along with four additional Illumina RNA‐seq data sets related to Bc‐grape berry interactions, were processed and simultaneously mapped against the PN40024 (12X.v2) and the *B. cinerea* DW1 genome assemblies (concatenated genomes). Mapped reads were counted and assigned to genes considering both the PN40024 VCost.v3 annotation and the BcDW1 genome annotation (GenBank GCA_000349525.1), and then imported into the Atlas tool (metadata found in Table S4).

**FIGURE 4:**
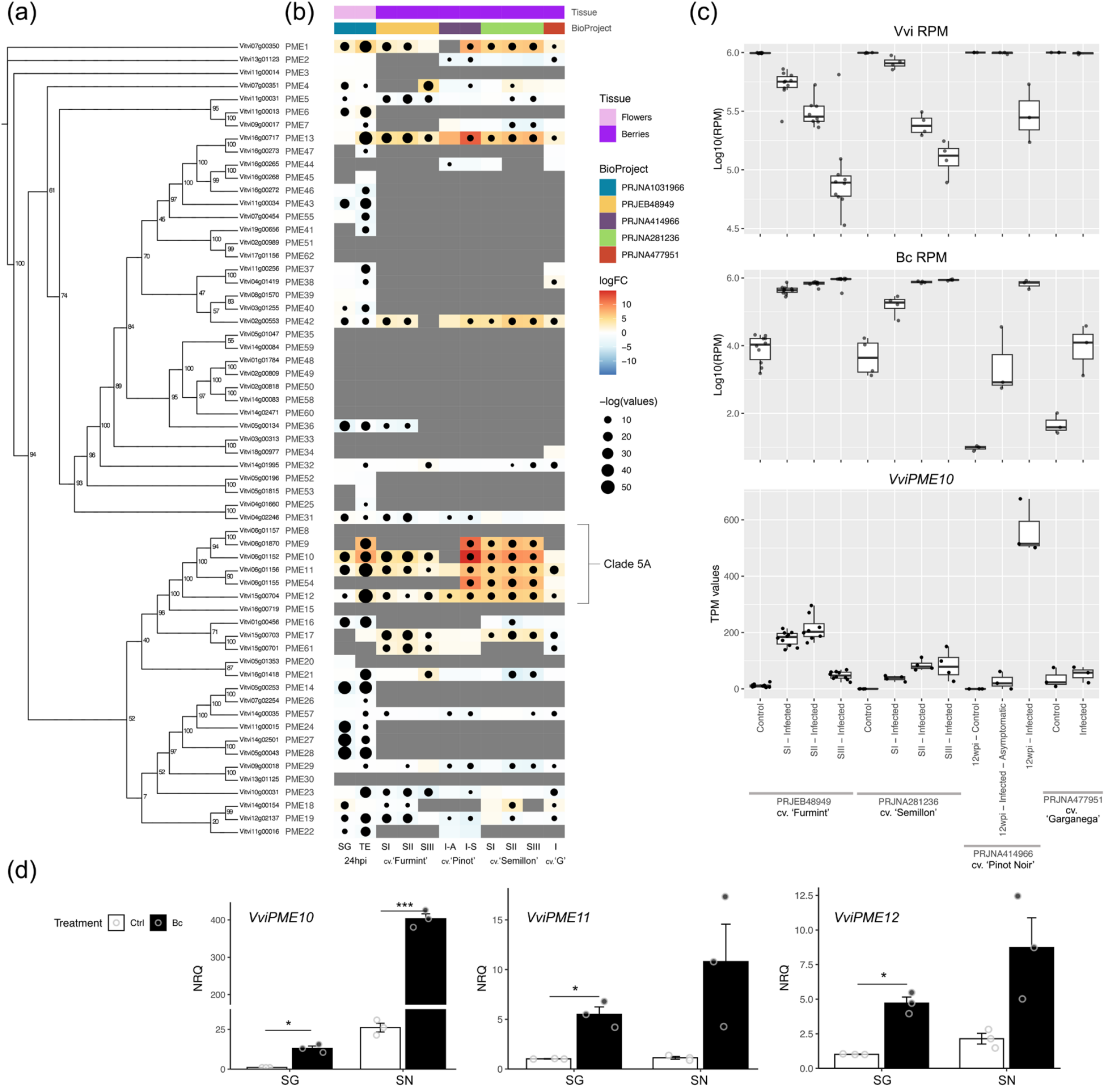
Clade 5A *PME* genes are highly induced during *B. cinerea* interactions in grapevine flowers and berries. (a) Phylogenetic tree of the *PME* genes identified in the *V. vinifera* PN40024 12X.2 reference genome (VCost.v3 annotation). A Maximum Likelihood tree was constructed using IQ‐TREE, supported by 1000 bootstrap replicates (Hoang et al. 2018; Jeong et al. 2015) and visualised with Figtree (http://tree.bio.ed.ac.uk/software/figtree/). Bootstrap values are shown next to the nodes. Members of clade 5A are highlighted. A complete Arabidopsis‐grapevine phylogenetic analysis is provided in Figure S2. (b) Co‐expression heatmap of *VviPME* genes derived from RNA‐seq experiments comparing *B. cinerea* ‐ vs. mock‐inoculated flowers of ‘Souvignier Gris’ (SG) and ‘Teroldego’ (TE) at 24 hpi, and from additional RNA‐seq data sets capturing *Bc*‐grapevine interaction in berries, queried via the Botrytis Stress Atlas Explorer tool (http://plantaeviz.tomsbiolab.com/vitviz/botrytis_atlas/). I‐A, infected asymptomatic; I‐S, infected symptomatic; SI, Stage I noble rot; SII, Stage II noble rot; SIII, Stage III noble rot. A summary of the datasets used is provided in Table S4. (c) Total abundance of normalised reads (RPM) mapped to both *V. vinifera* and *B. cinerea* genomes in symptomatic, asymptomatic, and control samples from the experiments shown in panel ‘b’. Mapping was performed using the PN40024 12X.v2 genome assembly (Canaguier et al. 2017) and the *B. cinerea* DW1 assembly (Blanco‐Ulate et al. 2013). TPM values for *VviPME10* in berry samples are also reported. (d) Expression levels of *PME10*, *PME11* and *PME12* in berry skins of ‘Souvignier Gris’ (SG) and ‘Sangiovese’ (SN) at 12 weeks post‐inoculation (wpi), measured by quantitative PCR (qPCR). Bars show normalised relative quantity (NRQ) in control (Ctrl) and *B. cinerea* ‐inoculated (Bc) samples. Expression values were calibrated to SG control levels and normalised using the reference genes *Actin* (*Vitvi04g01613*), *ATP16* (*Vitvi03g00055*), *Ubiquitin* (*Vitvi19g00434*) and *EF1α* (*Vitvi06g00319*). Data are presented as mean ± SD (*n* = 3). Asterisks (*) denote statistically significant differences between mock and *B. cinerea* ‐treated samples (Student's *t*‐test, * *p* ≤ 0.05, *** *p* ≤ 0.001).

Gene expression profiles of *PME* genes across the different experiments were retrieved through the Botrytis Stress Atlas Explorer. Using this platform, several *PME* genes were observed as differentially expressed in both floral and berry tissues following infection. Co‐expression heatmaps are displayed alongside a simplified *VviPME* phylogenetic tree (Figure 4a,b), while the complete *Vitis–Arabidopsis* phylogeny is presented in Figure S2. Notably, while a general downregulation of *PMEs* was observed upon Bc infection, *PME8, PME9, PME10, PME11, PME12* and *PME54* (Clade 5A *PMEs*) were consistently and significantly induced, regardless of cultivar or infection stage, with *PME10* being the most highly induced. This finding suggests that specific PME family members play a role in the response to *B. cinerea* , with PME10 appearing to have a predominant function. Despite *PME10* expression being more strongly induced in the susceptible cultivar ‘Teroldego’ than in the resistant hybrid ‘Souvignier Gris’ following infection, its basal expression level was considerably higher in the resistant genotype (Figure S4e). Further exploration of datasets within the Botrytis Stress Atlas revealed that total grapevine reads decline in proportion to infection severity, while *B. cinerea* reads increase (Figure 4c). Despite this reduction in host transcript abundance, *PME10* expression continues to rise as infection progresses, as observed in both ‘Furmint’ and ‘Semillon’, and in the asymptomatic versus symptomatic comparisons in infected ‘Pinot Noir’. Additionally, *PME10, PME11* and *PME12* were significantly induced in berry skins of both SG and SN upon infection, with the induction being more pronounced in the susceptible genotype (Figure 4d).

### 2.7 *PME10* Knockout Lines Showed an Impaired Induction of PME Activity and Higher Susceptibility When Infected With *B. cinerea*

**FIGURE 5:**
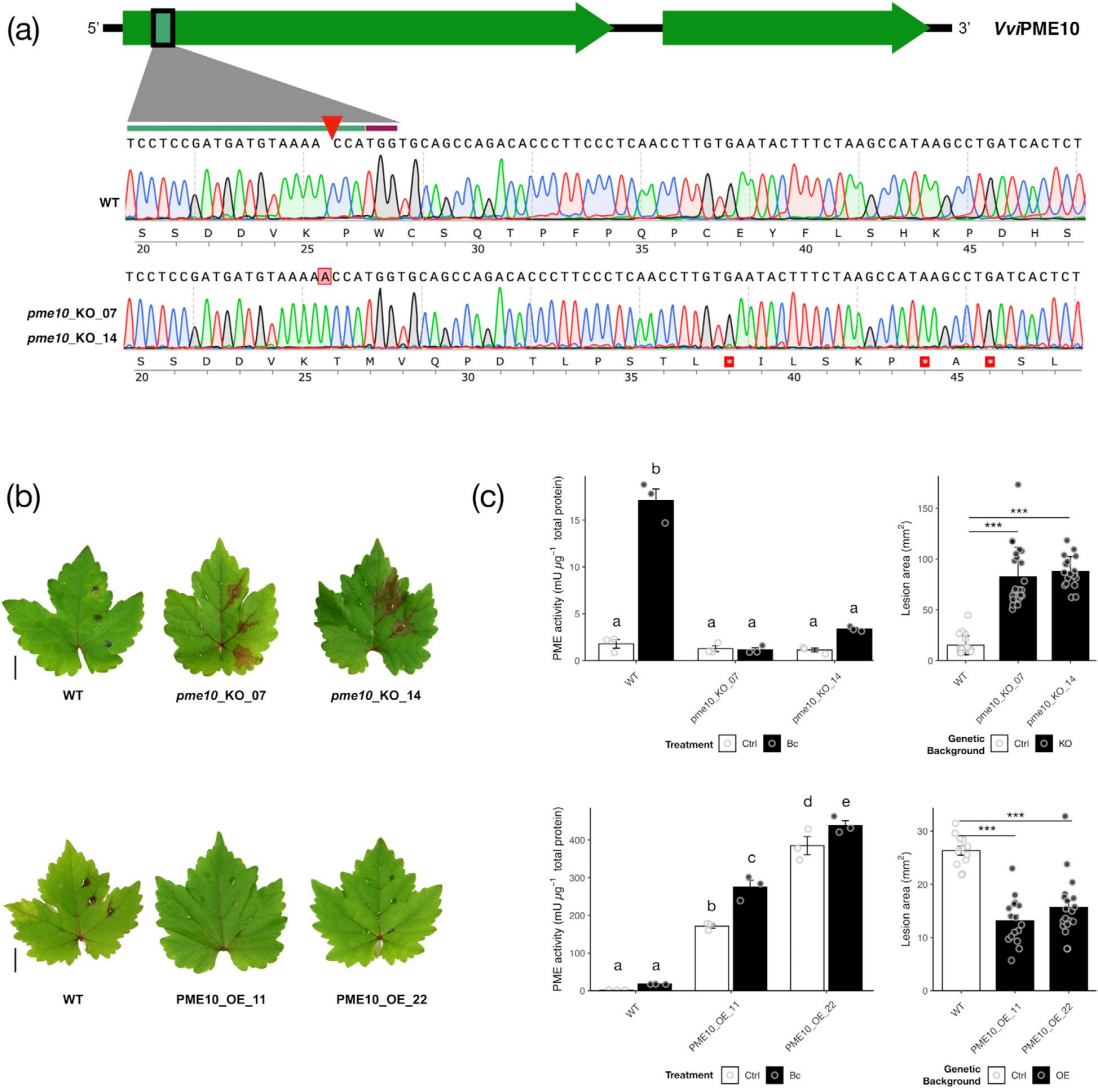
Grapevine *pme10* mutants are defective in PME induction and more susceptible to *B. cinerea* , whereas *PME10* overexpressing lines show elevated PME activity and increased resistance to the fungus. (a) Chromatograms of the on‐target *PME10* region in wild‐type (WT) and the *pme10*_KO_07 and *pme10_*KO_14 lines, all in the cv. ‘Sugraone’ genetic background. The position of the mutations, type of mutation (+A), and the resulting premature stop codons caused by the frameshift are highlighted in red. (b) Representative images of *Bc*‐infected leaves from WT and transgenic lines at 5 days post‐inoculation (dpi). Scale bar = 1 cm. (c) Left: Quantification of PME activity in protein extracts from uninfected and infected leaf sections of WT and transgenic lines. Bars represent the standard error of the mean (SEM). Different letters indicate statistically significant differences according to ANOVA followed by Tukey's test (*p* < 0.05). Right: Estimation of disease severity based on the mean leaf area (mm^2^) covered by brown necrotic lesions, measured on the left side of the leaf midrib. Bars indicate the standard error of the mean (SEM). Statistical significance was estimated using a *t*‐test (***adjusted *p*‐value ≤ 0.001).

To investigate the role of *PME10* in the response to *B. cinerea* infection, a reverse genetic approach was employed. Knockout (KO) lines for *PME10* were generated using CRISPR/Cas9‐mediated mutagenesis via *Agrobacterium tumefaciens* ‐mediated stable transformation of embryogenic callus derived from the highly transformable *V. vinifera* genotype cv. ‘Sugraone’. Among 23 regenerated lines, 18 carried mutations at the target site, as revealed by on‐target Illumina sequencing (Figure S5). The most frequent mutation was a single‐base insertion. Lines *pme10*_KO_07 and *pme10*_KO_14, both harbouring a 1 bp insertion (+A), resulting in a frameshift and premature stop codon (Figure 5a), were selected for further phenotypic characterisation. Four putative off‐targets were predicted in the *V. vinifera* PN40024 reference genome (Table S5) that correspond to the *PME* genes located within the same genomic region on Chromosome 6 as *PME10*, and belonging to the same clade (Clade 5A; Figures S2 and S6a). Notably, Sanger sequencing revealed that only off‐target 1 (*PME54*) carried a mutation in the line *pme10*_KO_07 (Figure S6b).

To assess whether the loss‐of‐function of *PME10* affects plant growth, we measured three phenotypic parameters in the greenhouse‐cultivated plants: height, internode length and total leaf area (Figure S7). No significant differences were observed between the *pme10* KO lines and controls, indicating that the mutation does not induce pleiotropic undesired effects impairing plant growth and development (Figure S7b). Next, the potential contribution of PME10 to the total PME activity induced by Bc was explored. Fully developed and healthy leaves were detached from independent replicates of the *pme10*‐KO‐07 and *pme10*‐KO‐14 lines and control plants and artificially inoculated with *B. cinerea* . After 5 days, brown necrotic symptoms were visible on the halves of the leaves inoculated with the fungus and were collected for further analysis (representative leaves from control plants and mutant lines are shown in Figure 5b). Total PME activity was quantified in both the infected and mock‐inoculated halves of the leaves from the mutants and control plants (Figure 5c). No significant differences in PME activity were observed between the uninfected mock‐inoculated mutants and control plants. Although no differences were observed in the mock inoculation, a significant induction (about 10‐fold) of PME activity was detected in control plants challenged with *B. cinerea* (Figure 5c). This evidence confirms that PME activity is induced in grapevine leaves upon Bc infection, as observed in other plants (Del Corpo et al. 2020; Lionetti and Métraux 2014). Interestingly, an impaired induction was observed in both the mutant lines that lack a significant induction of PME activity when challenged with *B. cinerea* . In particular, the results obtained with line *pme10*‐KO‐07 are remarkable. Indeed, in this line, the total absence of PME activity induction at 5 days post‐inoculation could be due to the mutation in the *PME54* gene (off‐target 1). These results indicate that PME10 is a pectin methylesterase that largely contributes to the induction of total PME activity in the grapevine against *B. cinerea*. This result agrees with the strong induction of the gene also in leaves upon Bc infection (Su et al. 2023).

To assess whether *PME10* loss‐of‐function affects grapevine susceptibility to Bc, disease severity, measured as lesion area (mm^2^), was evaluated on the leaves (Figure 5c). The lesion area was significantly larger in the *pme10*‐KO‐07 and *pme10*‐KO‐14 mutants, with an increase of 7.7‐ and 8.1‐fold, respectively, compared with control plants. A correlation between resistance level and PME activity was observed across all genotypes analysed. These results suggest that PME10 has a strong influence on PME activity and thus on grapevine's resistance to *B. cinerea* .

### 2.8 *PME10* Overexpressing Lines Showed High PME Activity, Restricting Botrytis Infection

To explore the effect of enhanced *PME10* expression on PME activity and Bc resistance, transgenic *V. vinifera* cv. ‘Sugraone’ lines overexpressing *PME10* were generated through *A. tumefaciens* ‐mediated stable transformation. Of the 20 regenerated plants, 18 showed the expected 574 bp amplicon. Two of the 18 transgenic lines, precisely lines *PME10* OE‐11 and *PME10* OE‐22, together with control plants, were further selected for the following phenotypic characterisation. The two lines showed a T‐DNA integration copy number close to 1, precisely 1.33 (*PME10* OE‐11) and 1.4 (*PME10* OE‐22). *PME10* expression analysis confirmed that both overexpressing lines showed higher levels of *PME10* transcript compared with the control plants (Figure S8b).

Concerning growth parameters, as shown for the PME KO lines (Figure S8a), no significant differences were found between *PME10* overexpressing and control plants (Figure S8c), confirming that PME10 is not involved in processes influencing plant development. Fully developed and healthy leaves were detached from independent replicates of the *PME10‐OE11* and *PME10‐OE22* lines and control plants and artificially inoculated with *B. cinerea* . After 5 days, brown necrotic symptoms were visible on the halves of the leaves inoculated with the fungus, mainly in the control plants, and therefore were collected for the following analysis. Representative leaves from control plants and overexpressing lines are shown in Figure 5b. Total PME activity was then compared between halves of the leaves from *PME10‐OE11* and *PME10‐OE22* lines and control plants, post mock‐ and Bc inoculation, respectively (Figure 5c). Mock‐inoculated *PME10‐OE11* and *PME10‐OE22* leaves showed a significantly higher PME activity (113.9‐ and 255.7‐fold, respectively) with respect to the control plants. This result confirms that *PME10* expression can strongly influence total PME activity in grapevine. A significant induction (about 11‐fold) of PME activity was detected in control plants challenged with *B. cinerea* . Interestingly, *PME10‐OE11* and *PME10‐OE22* lines showed lower inductions of Bc‐induced PME activity (1.6‐ and 1.1‐fold, respectively) with respect to their mock‐inoculated control. Since the results related to the *pme10* mutants indicate that no other PME isoform contributes to PME activity induction, the induction observed could be related to the stimulation of the *PME10* endogenous promoter. These results also suggest that the level of PME activity required for defence is already covered by the overexpression of the *PME10* transgene. Subsequently, the susceptibility of *PME10‐OE* lines to *B. cinerea* was evaluated. The local symptoms of the fungus were quantified in both the *PME10‐*OE lines and control plants. Interestingly, the area of the lesions produced by the fungus in *PME10‐OE11* and *PME10‐*OE22 was significantly lower (by 2 and 1.7‐folds, respectively) compared with the control (Figure 5c).

### 2.9 The *B. cinerea* ‐Induced WRKY03 Transcription Factor Directly Regulates *PME10* Expression

**FIGURE 6:**
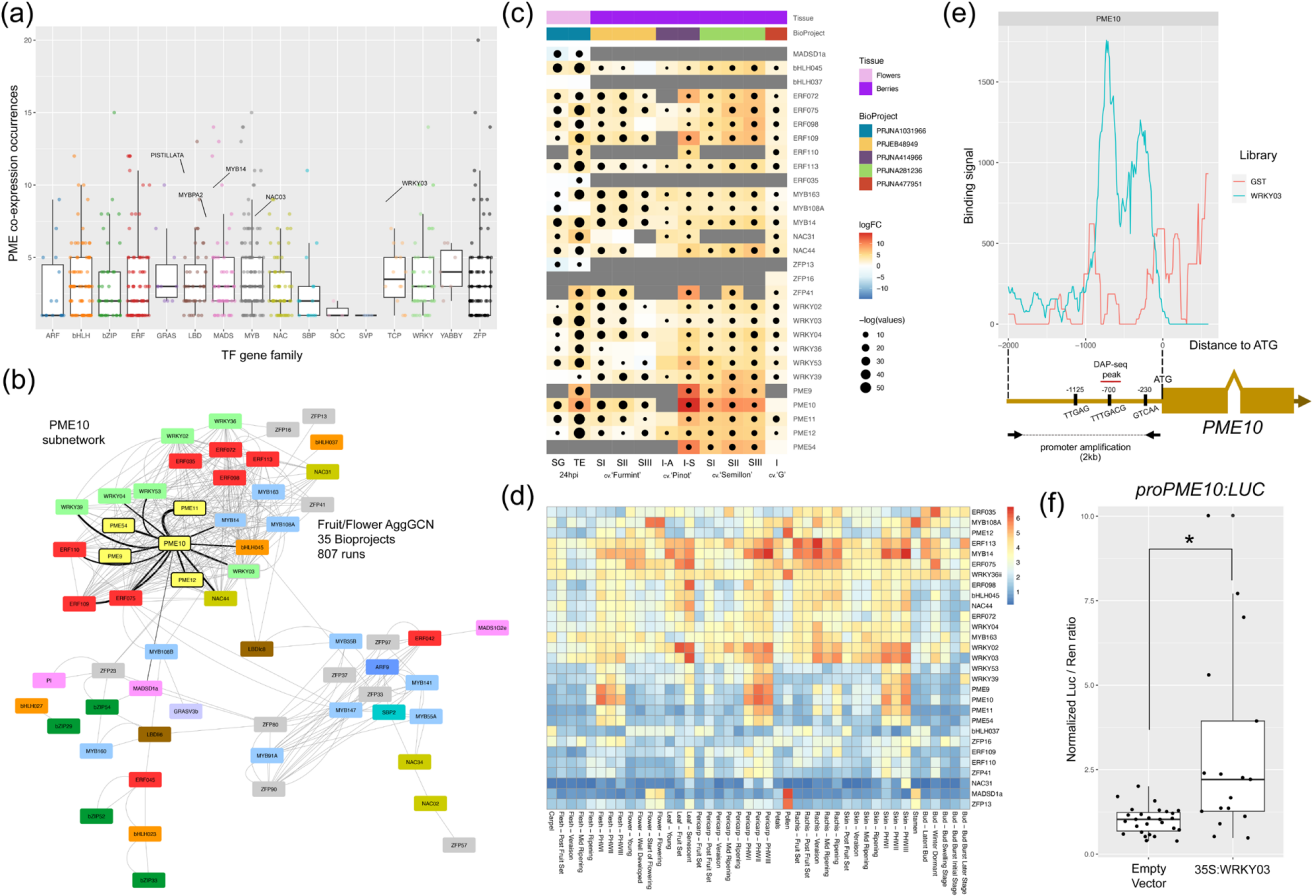
*Botrytis cinerea‐*induced WRKY03 transcription factor activates *PME10* expression. (a) Co‐expression analysis of each catalogued Transcription factor (TF) gene with the entire *PME* gene family. Sixteen TF classes are defined in grapevine (catalogue v2 found at Grapedia; https://grapedia.org/genes/). Dots indicate the number of *PME* genes co‐expressed with each TF, based on tissue‐independent gene co‐expression networks (GCNs), each comprising the top 1% of correlated genes (420 genes; Orduña et al. 2022, 2023). Previously characterised TFs found in the top quartile are highlighted. (b) Tissue‐specific co‐expression relationships in fruit and flower GCNs between PME‐Clade5A genes and the 62 topquartile TFs identified in (a). Three distinct clusters are observed, with the upper subnetwork designated as the ‘PME10 subnetwork’. Node distances were calculated using the d3Network package based on attraction metrics, which incorporate edge number and weight (default node repulsion mode). Visualisation was performed using Cytoscape (ver: 3.10.1, Shannon et al. 2003). (c) Expression profiles of the *PME10* subnetwork genes (plus *MADS1a*) during *B. cinerea* interaction in grapevine flowers and berries, shown as a co‐expression heatmap. Data are derived from RNA‐seq comparisons between *B. cinerea* ‐ and mock‐inoculated flowers of ‘Souvignier Gris’ (SG) and ‘Teroldego’ (TE) at 24 hpi, as well as from additional berry datasets queried through the Botrytis Stress Atlas Explorer (http://plantaeviz.tomsbiolab.com/vitviz/botrytis_atlas/). I‐A, infected asymptomatic; I‐S, infected symptomatic; SI, Stage I noble rot; SII, Stage II noble rot; SIII, Stage III noble rot. A summary of the RNA‐seq experiments imported in the tool is provided in Table S4. (d) Expression profiles of the genes across various grapevine tissues and different developmental stages by querying the VitViz Corvina Atlas (http://plantaeviz.tomsbiolab.com/vitviz/corvina_atlas/). Expression values are presented on a logarithmic scale and correspond to robust multiarray average (RMA) normalised values. (e) Top panel: WRKY03 DNA‐binding density surrounding the *PME10* transcription start site (TSS), plotted from −2 kb to +600 bp. The negative control corresponds to an input library generated using an empty GST‐HALO vector. Bottom panel: ositions of all predicted WRKY03 binding sites relative to the *PME10* ATG start codon. (f) Activation of the *PME10* promoter by WRKY03 assessed via dual luciferase reporter assay (DLRA) in *N. benthamiana* leaves. LUCIFERASE (LUC) activity was normalised to RENILLA (REN) levels. Asterisk denotes statistically significant differences (*p* < 0.05, *t*‐test).

To identify potential transcriptional regulators associated with *PME* genes, particularly those involved in *PME10* activation in response to *B. cinerea* infection, we performed a gene co‐expression analysis focused on the 16 transcription factors (TFs) families previously catalogued in grapevine (catalogue v2, Grapedia; https://grapedia.org/genes/). By mining aggregated gene co‐expression networks (GCNs) generated from extensive transcriptomic data (Orduña et al. 2022, 2023), we identified 11 TF families exhibiting more than eight PME co‐expression events within their top quartile, resulting in a set of 62 candidate TFs (Figure 6a). From this, we constructed a network of clade 5A *PME* genes and these 62 TFs, using flower and fruit RNA‐seq data sets, spanning 35 BioProjects and 807 runs. Within this network, a *PME10*‐centred subnetwork emerged, connecting 10 out of 23 TF regulators directly to *PME10*: *WRKY03, WRKY04, WRKY53, WRKY39, MYB14, NAC44, ERF75, ERF109, ERF110* and *bHLH045*. Additionally, *MADS1a* exhibited a direct co‐expression association with *PME10*, despite not being part of the PME subnetwork (Figure 6b). Most genes within the *PME10* subnetwork showed strong transcriptional modulation during *B. cinerea* infection (Figure 6c). Notably, *WRKY03*, *WRKY53*, *MYB14* and *NAC44* have been previously identified as key regulators of disease resistance through the control of stilbene biosynthesis, acting at different hierarchical levels with distinct combinatorial effects (Vannozzi et al. 2018; Wang et al. 2024; Wu et al. 2022). Among these, WRKY03 was particularly highlighted for its role in the response to infection. Expression profiling of *PME10* subnetwork members and *MADS1a* in berry and leaf tissues using the ‘Corvina’ Atlas Explorer (http://plantaeviz.tomsbiolab.com/vitviz/corvina_atlas/), revealed strong co‐expression of *PME10* with stilbenoid regulators *WRKY03* and *MYB14* during postharvest withering (i.e., over‐ripening) stages (Figure 6d). This finding aligns with previous reports identifying pectinesterase and stilbene synthase transcripts as biomarkers of withered berries (Fasoli et al. 2012), emphasising the importance of cell wall modification and resveratrol biosynthesis as key processes during the withering phase (Versari et al. 2001; Zamboni et al. 2008).

Among the transcription factors (TFs) identified in the PME10 subnetwork, only MYB14 (Orduña et al. 2022) and WRKY03 (SRA study PRJNA1199911) have been previously studied using DNA Affinity Purification Sequencing (DAP‐seq), a genome‐wide approach for identifying TF binding sites at the genome scale and inferring gene regulation. While *PME10* was absent from the bound targets of MYB14 and its closest homologue MYB15, reanalysis of public DAP‐seq data for *WRKY03* revealed a binding site located approximately 700 bp upstream of the *PME10* ATG start codon (in total, 62 WRKY03‐binding events on *PME* genes were identified, Table S6). Using the 21 nt *WRKY03* letter‐probability matrix derived from the top 600 best‐scoring peaks (Data S1) (with the core sequence TTGAC), we identified two additional putative binding sites at −230 bp and −1125 bp upstream of *PME10*. All three sites aligned with enriched binding signals observed in the DAP‐seq dataset (Figure 6e).

To validate the interaction of WRKY03 in the *PME10* promoter, we performed DAP‐qPCR using newly generated *WRKY03* (*n* = 3) and input (*n* = 3) DAP‐seq libraries, confirming significant enrichment at the‐1125 bp site and at the DAP‐seq identified peak centre (Figure S9). Furthermore, a dual luciferase reporter assay demonstrated that *WRKY03* strongly activates *PME10* expression upon co‐infiltration into tobacco leaves (a 2000 bp fragment of the *PME10* promoter, including the 5′ UTR, was fused to the *LUC* reporter gene; Figure 6f). Together, these results establish *WRKY03* as a direct transcriptional regulator of *PME10*, linking this defence‐related TF to PME‐mediated cell wall remodelling during *B. cinerea* infection.

## 3 Discussion

Many plant species induce local pectin methylesterase (PME) activity as part of their response to pathogen attack (Del Corpo et al. 2020). Consequently, increased PME activity is a hallmark of plant immunity and should be closely examined in the context of plant–pathogen interactions. In *Arabidopsis*, PME activity is tightly regulated post‐transcriptionally during immune responses to the necrotrophic fungus *B. cinerea* , particularly among Group II PMEs that contain a PRO or PMEI‐like domain. This regulation is mediated by subtilases (SBTs) and PME inhibitors (PMEIs), which modulate the activation and activity of these enzymes (Coculo et al. 2023; L'Enfant et al. 2015).

*Botrytis cinerea* is a broad‐host‐range necrotroph responsible for severe pre‐ and post‐harvest rots in over 200 plant species worldwide (Dean et al. 2012). The plant cell wall, together with the cuticle, forms the first physical and biochemical barrier against fungal invasion (Malinovsky et al. 2014). During infection, *B. cinerea* secretes its own arsenal of CWMEs, which act as key virulence factors facilitating host colonisation (Blanco‐Ulate et al. 2014). Simultaneously, the breakdown of the host cell wall can contribute to increased susceptibility (Cantu et al. 2008).

In this study, we investigated whether variation in PME activity and gene expression during berry development and upon *B. cinerea* infection contributes to grapevine susceptibility or resistance. This question is especially relevant considering that ripening‐associated softening of grape berries, a process characterised by extensive cell wall remodelling, coincides with heightened vulnerability to fungal infection. Indeed, specific members of grapevine CWME gene families are upregulated during fruit softening and may serve as susceptibility factors during the grape– *B. cinerea* interaction (Malacarne et al. 2024).

### 3.1 PME Activity Contributes Positively to Grapevine Immunity Against *B. cinerea*

The use of grapevine genotypes with contrasting susceptibility to Bc provided valuable insights into the role of cell wall (CW) composition and remodelling in the grapevine‐Bc interaction. Analysis of flower and berry tissues revealed no major differences between the tolerant genotype ‘Souvigner Gris’ and the susceptible *V. vinifera* cultivars ‘Teroldego’ and ‘Sangiovese’, both before and after Bc infection. This suggests that differences in resistance are not attributable to variations in CW polysaccharide content. As expected, galacturonic acid (Gal A), the major component of homogalacturonan (HG), was the predominant monosaccharide. The detection of arabinose, fucose, galactose, glucose, rhamnose, mannose and xylose further indicated the presence of other polysaccharides, such as rhamnogalacturonan and hemicelluloses (Harholt et al. 2010). These findings are novel, given the limited previous reports on CW composition in grapevine tissues across cultivars and infection conditions. Notably, they differ from those of (Weiller et al. 2021), who reported a decrease in GalA and an increase in glucuronic acid in berries of cultivars with differing susceptibility to Bc at 6 and 12 days post‐inoculation. These discrepancies may reflect differences in tissue type, developmental stage or timing of sampling.

Upon Bc infection, SG consistently showed a significant induction of PME activity compared with the susceptible genotypes, TE (in flowers) and SN (in berries). This increased PME activity in the tolerant genotype is associated with a reduction in the degree of CW methylesterification, a hallmark of pectin remodelling observed in other species (Lionetti et al. 2012). Interestingly, PME induction occurred as early as 24 h post‐inoculation (hpi) in SG flowers during the first experimental season but was delayed until 96 hpi in the second season. We interpret this delay as likely resulting from differences in the inoculum's physiological state or in the SG plants' age (1 year older in the second season). This age difference may have led to immune priming or developmental changes that altered the timing of the response. Despite this temporal variation, both experiments consistently showed a significant induction of PME activity in the resistant genotype upon infection, reinforcing the conclusion that PME activity contributes to grapevine resistance against Bc, in line with observations in Arabidopsis (Del Corpo et al. 2020; Coculo et al. 2023).

Functionally, pectin demethylesterification by PMEs in the primary CW (Mouille et al. 2007) enables the action of PGs and pectate lyases (PLs), which generate demethylesterified OGs that act as DAMPs, triggering plant immune responses (Osorio et al. 2008, 2011). PME activity also leads to methanol (MeOH) release, a volatile signal that functions as an alarm signal within interplant communication (Hann et al. 2014). These mechanisms support a model in which PME activity is not merely a passive response but a critical component of early defence against *B. cinerea* .

### 3.2 *PME10* Is a Pectin Methylesterase Responsible for the PME Activity That Confers Resistance to *B. cinerea*

To determine which PME isoforms contribute to the PME activity associated with resistance to *B. cinerea* , we examined transcriptomic differences between control and Bc‐infected tissues. Among the 62 annotated PME genes in grapevine, our analysis aimed to identify those most strongly associated with the induced PME activity observed during infection. RNA‐seq transcriptomic analysis of flower samples from the tolerant genotype ‘Souvigner Gris’ and the susceptible ‘Teroldego’ revealed a pronounced transcriptional response to Bc in TE, particularly at early infection stages. This extensive response, marked by the upregulation of numerous stress‐related genes, likely reflects an ultimately ineffective attempt to restrict pathogen progression. By contrast, SG exhibited a more limited transcriptional response, suggesting that its resistance relies on preformed or rapidly activated defences requiring less *de novo* transcriptional reprogramming. Among the *PME* genes, most were downregulated upon infection, yet a subset, *PME8, PME9, PME10* and *PME11*, was significantly upregulated, especially in the resistant genotype.

To expand this observation, we integrated gene expression data from multiple publicly available RNA‐seq data sets covering diverse cultivars and experimental conditions involving Bc infection of berries. These data were consolidated into a new open‐access visualisation tool, the Botrytis Stress Atlas Explorer, enabling cross‐experiment comparisons of *PME* gene expression (available at http://plantaeviz.tomsbiolab.com/vitviz/botrytis_atlas/). This meta‐analysis confirmed that the same *PME* genes highly induced in flowers, particularly *PME10*, were also consistently upregulated in infected berry tissues across cultivars, regardless of genetic background or infection stage. Among them, *PME10* emerged as the most strongly and consistently induced gene, showing robust expression upon Bc infection in both flowers and berries. Notably, *PME10* is also highly expressed in ripe and postharvest withered berries under noninfectious conditions, indicating a potential dual role in fruit maturation and pathogen defence. Based on these findings, we performed further characterisation to define the functional contribution of *PME10* to the grapevine defence response.

To functionally validate the role of PME10 in grapevine defence against *B. cinerea* , we generated PME10 knockout lines using the CRISPR/Cas9 system, a technique previously applied to dissect gene function in response to both biotic (Giacomelli et al. 2023; Wan et al. 2020) and abiotic (Clemens et al. 2022) stresses. In parallel, we also developed PME10‐overexpressing lines to complement the mutant analysis and further corroborate PME10's contribution to disease resistance. This dual approach allowed us to assess the impact of both loss and gain of PME10 function on PME activity and susceptibility to *B. cinerea* .

*PME10* knockout mutants and overexpressing lines showed no developmental changes, suggesting that *PME10* does not affect growth or plant fitness and could be involved specifically in the defence response. Knocking out the PME10‐PRO region, which has very little homology within the gene family, was highly effective. *PME10* loss‐of‐function mutants showed reduced PME activity and were more susceptible to *B. cinerea* infection. Consistently, overexpression of *PME10* led to high PME activity, conferring immunity to the fungus and reducing disease severity. This provides evidence that VviPME10 functions as a pectin methylesterase contributing to the induction of total PME activity in leaves in response to *B. cinerea* , mirroring the role of AtPME17 in *Arabidopsis* immunity against the same fungus (Del Corpo et al. 2020). In fact, *VviPME10* is an ortholog of *AtPME17*.

The results obtained here suggest that VviPME10 is a resistance factor that could initiate a defence response even before infection, similar to AtPME17 (Del Corpo et al. 2020) and FaPE1 (Osorio et al. 2011) which are homologues involved in this process in other species. Indeed, PMEs can trigger plant immunity in different ways (Del Corpo et al. 2024). PMEs generate negatively charged HG regions that promote Ca2+–mediated crosslinking, strengthening the cell wall and preventing pathogen entry (Del Corpo et al. 2020). They also release demethylesterified OGs and methanol, whose role is to trigger responses ranging from cell wall reinforcement to the synthesis of defence molecules such as phytoalexins, pathogenesis‐related (PR) proteins and reactive oxygen species (Ferrari et al. 2013; Hann et al. 2014; Osorio et al. 2011). On the other hand, PME activity, which increases the level of demethylesterified pectin, loosens the cell wall, favouring the colonisation of necrotrophs. For instance, the *AtPME3* gene is a susceptibility factor for initial colonisation (Raiola et al. 2011).

### 3.3 WRKY03 Directly Activates *PME10* Expression in Response to *B. cinerea*

To gain insight into the regulatory mechanisms controlling *PME10* expression during *B. cinerea* infection, we first explored the *PME10* gene co‐expression network, which identified several candidate transcription factors (TFs), including WRKY03, WRKY53, MYB14 and NAC44. These TFs have previously been implicated in the regulation of stilbene biosynthesis and associated disease resistance responses in grapevine (Vannozzi et al. 2018; Wu et al. 2022; Wang et al. 2024). We focused on WRKY03 (also referred to as WRKY8 in Jiang et al. (2019)), a gene previously proposed to play a role in *B. cinerea* response (Guo et al. 2018). Three putative WRKY03 binding sites were identified in the *PME10* promoter, one of which overlapped with a DAP‐seq peak centre. DAP‐qPCR assays confirmed significant enrichment of WRKY03 binding at positions −1125 bp and at the DAP‐peak centre. A subsequent transactivation assay further confirmed that WRKY03 activates *PME10* promoter activity. These results support a model in which WRKY03 directly activates *PME10* expression in response to fungal infection, potentially linking cell wall remodelling through PME10 with stilbene‐mediated defences, thereby contributing to the restriction of pathogen progression.

## 4 Conclusions

Taken together, our findings demonstrate that PME activity, primarily mediated by PME10, plays a pivotal role in shaping grapevine immunity against *B. cinerea*. PME10‐driven pectin demethylesterification not only contributes to cell wall reinforcement as a physical barrier but also appears to function in signalling pathways that enhance pathogen perception and immune activation. We propose a regulatory network in which *PME10* expression is modulated in response to *B. cinerea* infection, with WRKY03 identified as a key upstream regulator. This transcription factor directly binds and activates the *PME10* promoter, providing mechanistic insight into how grapevine transcriptional responses integrate cell wall remodelling with defence signalling. Our work positions PME10 as a novel molecular marker of *B. cinerea* resistance in grapevine and a promising target for crop protection strategies. These findings offer a foundation for the development of biologically based approaches to control Botrytis bunch rot, a major threat to global viticulture. Targeting PME10 or its associated pathways could contribute to more resilient grapevine cultivars, aligning with the goals of sustainable viticulture by reducing reliance on chemical fungicides and fostering environmentally sound agricultural practices.

## 5 Experimental Procedures

### 5.1 Plant Material

#### 5.1.1 Grapevine Genotypes for Artificial Inoculation With *B. cinerea*

Three grapevine genotypes were selected for artificial infection with *B. cinerea* across two independent experiments conducted in consecutive seasons (2022 and 2023): ‘Souvigner Gris’ (SG), a *Vitis* interspecific hybrid (‘*Seyval*’ × ‘*Zähringer*’), and two *V. vinifera* cultivars, ‘Teroldego rotaliano’ (TE) and ‘Sangiovese’ (SN). Genotype selection was based on previously published resistance phenotypes (Rahman et al. 2018) and on 3 years of phenotypic evaluation of the Fondazione Edmund Mach (FEM) grapevine germplasm collection. These phenotypic data were standardised and ontologised in a dedicated database currently under development (Vezzulli et al. 2022). Plants were propagated by grafting wood cuttings onto KOBER 5BB rootstock, and grown in 32 L pots containing a soil mixture of peat, coconut, pumice and clay. All plants were maintained under a tunnel at the Giaroni experimental field (46°18′ N, 11°13′ E) of FEM.

### 5.2 *PME10* Knockout and Overexpressing Lines

#### 5.2.1 *PME10* Cloning and Overexpression

The *PME10* gene was amplified from cDNA obtained from senescent *V. vinifera* PN40024 leaves. RNA was extracted from 100 mg of homogenised senescent leaves (Chang et al. 1993). Total RNA was treated with DNAse I and first‐strand cDNA was synthesised using SuperScript III Reverse Transcriptase (Thermo Scientific), according to the manufacturer's instructions. To overexpress *PME10*, full‐length CDS (*Vitvi06g01152*) was amplified from cDNA using Phusion Hot Start II DNA Polymerase (Thermo Scientific), cloned into pENTR/D‐TOPO and recombined into pK7WG2 using Gateway LR Clonase II enzyme mix (Thermo Scientific). The cloned sequence was confirmed as *PME10* and used for *A. tumefaciens* transformation.

#### 5.2.2 *PME10* Knockout

A sgRNA specific for *PME10* (5′‐CCTCCGATGATGTAAAACCA‐3′) was designed to target the region encoding its PMEI‐like domain (PRO part), which has low homology within the gene family. The sgRNA sequence was designed using three online tools: (i) CRISPR‐P v2 (Liu et al. 2017), (ii) CRISPOR (Concordet and Haeussler 2018) and (iii) CHOPCHOP (Labun et al. 2019). The *PME10* sequence was used as input to select the target site, using the default settings for SpCas9 (NGG as PAM) and AtU6.26 promoter. The target site was confirmed in the *V. vinifera* Sugraone genome scaffold containing the target region (kindly provided by the Computational Biology Unit at FEM). The secondary structure and sequence of the selected sgRNA for *PME10* were confirmed using Vienna RNA Package 2.0 (Lorenz et al. 2011). The gRNA was cloned into the pD764‐PME10‐sgRNA2 plasmid using BsmBI. It contains the SpCas9‐WT coding sequence, and the expression of the gRNA is controlled by the *A. thaliana* U6.26 promoter.

#### 5.2.3 Stable Gene Transfer Experiments

Binary vectors for the CRISPR/Cas9 knockout (KO) or the overexpression (OE) of the *PME10* gene were transformed into *A. tumefaciens* EHA105 strain, harbouring the pCH32 helper (Hamilton 1997) using the protocol by (Wise et al. 2006). *A. tumefaciens* stable gene transfer was performed according to (Dalla Costa et al. 2022).

All the experimental procedures for molecular analyses, acclimation and growth characterisation of *PME10* OE and KO lines are available in Methods S1.

### 5.3 Bc Artificial Inoculation Assays

#### 5.3.1 *B. cinerea* Inoculum Preparation

*Botrytis cinerea* B05.10 was grown on Potato Dextrose Agar (PDA) plates with 20 mL/L tomato puree. The Bc suspension for the inoculum was prepared using 10‐day‐old *Bc* B05.10 plates according to (Vega et al. 2015). Conidia were suspended in Gamborg B5‐2% glucose medium and adjusted to 2 × 10^5^ conidia/mL in the same medium with 10 mM KH_2_PO4/K_2_HPO4, pH 6.4.

#### 5.3.2 *B. cinerea* Artificial Inoculation of Flowers of Different Grapevine Genotypes

Inflorescences at full cap‐fall stage (EL25/26, according to Eichhorn and Lorenz (1977)) of the three different grapevine genotypes were artificially inoculated following the procedure described in Methods S2.1. Two independent experiments were performed and samples were collected at 24‐h post‐inoculation (hpi) (Season 1), and at 24 and 96 hpi and at 12‐week post‐inoculation (wpi) (Season 2), and snap‐frozen until use (Figure 1).

#### 5.3.3 *B. cinerea* Artificial Leaf Inoculation Assays of KO and OE Lines

Leaves from the *VviPME10* KO and OE lines, as well as the control plants, were inoculated using the protocol by (Vega et al. 2015) and following the procedure detailed in Methods S2.2.

### 5.4 Biochemical and Immunohistochemical Analyses

#### 5.4.1 Determination of Methyl Ester Content and Monosaccharide Composition in CW of Flower and Berry Skin Samples

Alcohol‐insoluble residue (AIR) extraction and CW composition were performed as previously described (Lionetti et al. 2017). The degree of methyl esterification in CW of *B. cinerea* ‐infected and mock‐inoculated flower and berry skin samples was assessed using a microplate‐adapted alcohol oxidase/acetylacetone method (Klavons and Bennett 1986).

#### 5.4.2 PME Activity Determination

Protein extracts were prepared by homogenising uninfected and infected grapevine tissues in a buffer containing 1 M NaCl, 12.5 mM citric acid, 50 mM Na_2_HPO_4_, 1% polyvinylpyrrolidone, 0.02% sodium azide, and protease inhibitor (1:100 v/v), pH 7.0. After shaking (3 h, 4°C) and centrifugation (15 000 × g, 15 min), protein concentrations in supernatants were measured by the Bradford assay using BSA as a standard. PME activity was evaluated using the PECTOPLATE assay (Lionetti 2015).

#### 5.4.3 Immunohistochemical Analysis

Samples for immunohistochemistry were immersed in FAA solution and vacuumed for 10 min. Following fixation, the tissue was dehydrated in ethanol using increasing concentrations and embedded in Paraplast Plus (Sigma‐Aldrich) as described in (Rojas et al. 2021).

The complete experimental procedures on biochemical and immunohistochemical analyses are in Methods S3.

### 5.5 Gene Expression Analyses

Flower samples collected at 24 h post‐inoculation (hpi) during the first experimental season were used for RNA‐seq analysis. RNA extraction, library preparation, sequencing and downstream data processing were performed as described in Methods S4.2. Summary statistics for RNA‐seq quality control and mapping are provided in Table S2, and the complete list of differentially expressed genes (DEGs) is available in Table S3. Berry skin samples collected at 12 weeks post‐inoculation (wpi) during the second experimental season were used for quantitative PCR (qPCR) analysis. The qPCR protocol is detailed in Methods S4.2. Gene expression levels were assessed using gene‐specific primers (Table S8), with *Actin* (*Vitvi04g01613*), *ATP16* (*Vitvi03g00055*), *Ubiquitin* (*Vitvi19g00434*) and *EF1α* (*Vitvi06g00319*) as reference genes for normalisation. Primer specificity was verified by melting curve analysis, performed during each qPCR reaction (Figure S10).

### 5.6 Bioinformatics

All computational analyses can be found in Methods S4.

### 5.7 *PME10* Promoter Analysis and Cloning, DAP‐Seq and DAP‐qPCR Analyses and Dual Luciferase Assay

The procedures related to these tasks can be found in Methods S5.

## Author Contributions

G.M. designed and supervised the study and acquired the funds. J.L. performed the genetics and physiological studies. D.C. and V. L. carried out the biochemical analyses. J.L. and A.S.P. performed the flower RNA‐seq analysis and the berry RNA‐Seq reanalysis. A.S.P. constructed the Botrytis Stress Atlas Explorer. B.R. contributed to artificial infection experiments. L.D.C. and M.M. collaborated with J.L. to generate transgenic plants. A.S.P., A.V. and J.T.M. carried out the DAP‐seq experiment and data analysis. M.‐B.T. performed the DAP‐qPCR experiments. J.L., C.Z. and G.A.P. performed the transient expression assays in tobacco leaves. G.M., J.L., V.L. and J.T.M. wrote the manuscript. All the authors discussed the results and read and approved the final manuscript.

## Conflicts of Interest

The authors declare no conflicts of interest.

## Supporting information


**Data S1.** Letter‐probability matrix for WRKY03 transcription factor binding sites (TFBS).

**Figure S1.** Monosaccharide compositions of cell wall extracts from flowers and berry skins of different grapevine genotypes.
**Figure S2.** Phylogenetic tree of *Arabidopsis thaliana* and *Vitis vinifera* Pectin Methyl Esterase (*PME*) genes.
**Figure S3.** Expression profiles of *PME* family genes across various grapevine organs and tissues at different developmental stages.
**Figure S4.** Summary of RNA‐seq results comparing Bc‐infected and control flowers of ‘Souvigner Gris’ (SG) and ‘Teroldego’ (TE) at 24 h post‐inoculation.
**Figure S5.** Summary of on‐target analysis of the *PME10* knockout (KO) lines.
**Figure S6.** Summary of the off‐target analysis of the *PME10* KO lines.
**Figure S7.** Phenotypic characterisation of *PME10* KO lines compared with control plants.
**Figure S8.** Phenotypic characterisation of *PME10* overexpressing (OE) lines compared with control plants.
**Figure S9.** WRKY03 DAP‐seq and DAP‐qPCR analyses of the WRKY03‐PME10 interaction.
**Figure S10.** Melting curve analysis during qPCR assays using primers for *PME10*, *PME11*, and *PME12*.
**Table S1.** Complete list of the 62 *PME* genes identified in the *V. vinifera* PN40024 reference genome.
**Table S2.** Summary of the Illumina read processing and mapping to the concatenated *V. vinifera* PN40024 12X.v2 and *B. cinerea* DW1 genome assemblies.
**Table S3.** Differentially expressed genes in ‘Souvigner Gris’ and *V. vinifera* ‘Teroldego’ flowers at 24 h post‐inoculation with *B. cinerea*.
**Table S4.** Metadata of publicly available RNA‐seq experiments on *B. cinerea* ‐grapevine berry interactions, included in the Botrytis Stress Atlas Explorer.
**Table S5.** Predicted *PME10* off‐target regions in *V. vinifera* ‘PN40024’ and ‘Sugraone’ genome assemblies.
**Table S6.** WRKY03‐binding events on *PME* genes detected by DAP‐seq analysis.
**Table S7.** *PME10* DAP‐seq qPCR conditions.
**Table S8.** List of primers used throughout the study.
**Methods S1.** Molecular analysis and acclimation procedures for PME10 OE and KO lines.
**Methods S2.** Detailed procedures for Bc artificial inoculation assays.
**Methods S3.** Detailed experimental procedures for biochemical and immunohistochemical analyses.
**Methods S4.** Computational and gene expression analysis workflows.
**Methods S5.** Detailed experimental procedures for DAP‐seq and DAP‐qPCR analyses, *PME10* promoter analysis and cloning, and dual luciferase assay.

## Data Availability

The Botrytis stress Atlas Explorer is available at the VitViz suite within the PlantaeViz Platform (http://www.plantaeviz.tomsbiolab.com/). The PME family and the biological role of PME10 studied here have been deposited in the Gene Reference Catalogue found at the Grape Genomics Encyclopedia portal (http://grapedia.org/). The raw RNA‐Seq read data were deposited in the NCBI Short Read Archive under the BioProject accession code PRJNA1031966.
