# Supplementary material for: PME10 Is a Pectin Methylesterase Driving PME Activity and Immunity Against Botrytis cinerea in Grapevine (Vitis vinifera L.)
Source: Plant Biotechnol J. 2025 Jul 29;23(11):4981–97. doi: 10.1111/pbi.70279 (PMC12576464; doi:10.1111/pbi.70279)
Supplement: Supplementary file 1 — Data S1. Letter‐probability matrix for WRKY03 transcription factor binding sites (TFBS). [file PBI-23-4981-s001.docx]

**Data S1.** **Letter-probability matrix for WRKY03 transcription factor binding sites (TFBS).**

## WRKY03 Transcription Factor Binding Motifs (TFBM)

Detected based on DAP-seq data on Cabernet franc young leaf.

### ALPHABET= ACGT

Strands: + -

### Background letter frequencies (from unknown source):

| A | 0.300 |
| --- | --- |
| C | 0.200 |
| G | 0.200 |
| T | 0.300 |

### Log-odds matrix: alength= 4 w= 21 E= 4.8e-814

| -17 | -12 | 66 | -36 |
| --- | --- | --- | --- |
| -47 | -12 | 82 | -28 |
| 19 | -23 | -39 | 16 |
| -41 | -23 | -5 | 45 |
| 36 | -21 | -43 | -3 |
| -16 | -25 | -23 | 39 |
| 34 | -22 | 16 | -42 |
| 76 | -72 | -22 | -58 |
| -52 | -18 | 23 | 34 |
| -4 | 66 | -18 | -48 |
| -161 | -689 | 183 | -68 |
| -1586 | -389 | -1586 | 172 |
| -1586 | -1586 | -1586 | 174 |
| -1586 | -1586 | 232 | -1586 |
| 174 | -1586 | -1586 | -1586 |
| -1586 | 232 | -1586 | -1586 |
| -547 | -50 | -1586 | 151 |
| -180 | -457 | -250 | 154 |
| -169 | -168 | -237 | 143 |
| -69 | -143 | 0 | 85 |
| -40 | -74 | 13 | 53 |

### Letter-probability matrix: alength= 4 w= 21 nsites= 593 E= 4.8e-814

| 0.266442 | 0.183811 | 0.317032 | 0.232715 |
| --- | --- | --- | --- |
| 0.215852 | 0.183811 | 0.354132 | 0.246206 |
| 0.342327 | 0.17032 | 0.153457 | 0.333895 |
| 0.22597 | 0.17032 | 0.193929 | 0.409781 |
| 0.384486 | 0.173693 | 0.148398 | 0.293423 |
| 0.268128 | 0.168634 | 0.17032 | 0.392917 |
| 0.379427 | 0.172007 | 0.224283 | 0.224283 |
| 0.505902 | 0.121417 | 0.172007 | 0.200675 |
| 0.209106 | 0.177066 | 0.234401 | 0.379427 |
| 0.291737 | 0.317032 | 0.177066 | 0.214165 |
| 0.097808 | 0.001686 | 0.713322 | 0.187184 |
| 0.0 | 0.013491 | 0.0 | 0.986509 |
| 0.0 | 0.0 | 0.0 | 1.0 |
| 0.0 | 0.0 | 1.0 | 0.0 |
| 1.0 | 0.0 | 0.0 | 0.0 |
| 0.0 | 1.0 | 0.0 | 0.0 |
| 0.006745 | 0.141653 | 0.0 | 0.851602 |
| 0.086003 | 0.008432 | 0.035413 | 0.870152 |
| 0.092749 | 0.062395 | 0.038786 | 0.806071 |
| 0.185497 | 0.074199 | 0.200675 | 0.539629 |
| 0.227656 | 0.11973 | 0.219224 | 0.43339 |
