# Supplementary material for: PME10 Is a Pectin Methylesterase Driving PME Activity and Immunity Against Botrytis cinerea in Grapevine (Vitis vinifera L.)
Source: Plant Biotechnol J. 2025 Jul 29;23(11):4981–97. doi: 10.1111/pbi.70279 (PMC12576464; doi:10.1111/pbi.70279)
Supplement: Supplementary file 2 — Figure S1. Monosaccharide compositions of cell wall extracts from flowers and berry skins of different grapevine genotypes. Figure S2. Phylogenetic tree of Arabidopsis thaliana and Vitis vinifera Pectin Methyl Esterase (PME) genes. Figure S3. Expression profiles of PME family genes across various grapevine organs and tissues at different developmental stages. Figure S4. Summary of RNA‐seq results comparing Bc‐infected and control flowers of ‘Souvigner Gris’ (SG) and ‘Teroldego’ (TE) at 24 h post‐inoculation. Figure S5. Summary of on‐target analysis of the PME10 knockout (KO) lines. Figure S6. Summary of the off‐target analysis of the PME10 KO lines. Figure S7. Phenotypic characterisation of PME10 KO lines compared with control plants. Figure S8. Phenotypic characterisation of PME10 overexpressing (OE) lines compared with control plants. Figure S9. WRKY03 DAP‐seq and DAP‐qPCR analyses of the WRKY03‐PME10 interaction. Figure S10. Melting curve analysis during qPCR assays using primers for PME10, PME11, and PME12. Table S1. Complete list of the 62 PME genes identified in the V. vinifera PN40024 reference genome. Table S2. Summary of the Illumina read processing and mapping to the concatenated V. vinifera PN40024 12X.v2 and B. cinerea DW1 genome assemblies. Table S3. Differentially expressed genes in ‘Souvigner Gris’ and V. vinifera ‘Teroldego’ flowers at 24 h post‐inoculation with B. cinerea. Table S4. Metadata of publicly available RNA‐seq experiments on B. cinerea ‐grapevine berry interactions, included in the Botrytis Stress Atlas Explorer. Table S5. Predicted PME10 off‐target regions in V. vinifera ‘PN40024’ and ‘Sugraone’ genome assemblies. Table S6. WRKY03‐binding events on PME genes detected by DAP‐seq analysis. Table S7. PME10 DAP‐seq qPCR conditions. Table S8. List of primers used throughout the study. Methods S1. Molecular analysis and acclimation procedures for PME10 OE and KO lines. Methods S2. Detailed procedures for Bc artificial inoculation assays. Metho [file PBI-23-4981-s002.zip › pbi70279-sup-0013-Methods.docx]

**fMethods S1. Molecular analysis and acclimation procedures for PME10 OE and KO lines.**

***S1.1 Molecular Analysis of Transgenic Regenerated Lines***

Genomic DNA was extracted from *in vitro* regenerated *PME10* KO and OE lines following the CTAB protocol (Allen *et al.*, 2006). The presence of T-DNA was evaluated by PCR. For the *PME10* KO lines, a 281 bp target amplicon was expected using sgRNA2-VviPME10-F and pCRISPR-RB-rev primers, while for the *PME10* OE lines, a 574 bp amplicon was expected when using a primer pair spanning the P35S and PME10 sequences (Table S8).

***S1.2 On/off-target Analysis of PME10 KO-lines***

The presence of a possible on-target mutation at the *PME10* target site was first assessed by Sanger sequencing of a 354 bp amplicon. Once the DNA mutation was confirmed, the samples were analysed using Illumina MiSeq amplicon sequencing. The target site was amplified by PCR using specific primers with Illumina adaptor overhangs (Table S8). After purification (Clean NGS Kit, Clean NA), the PCR products were used to prepare Illumina libraries, sequenced on the MiSeq platform. The WT genotype was used as a reference for mutation analysis. Modification at the target cleavage site was analysed using CRISPResso2, version 2.1.3 (Clement *et al.*, 2019) by aligning Illumina reads of putatively edited lines to those of WT. Mutations in the cleavage target site (insertion, deletion, and substitutions) were quantified accordingly and the protein modifications were predicted *in silico*. Predicted off-targets for the selected guide RNAs were identified using CRISPOR (Concordet and Haeussler, 2018) and *V. vinifera* PN40024 12X.v2 genome assembly as reference genome (Canaguier *et al.*, 2017). The identified putative off-target sites were checked in the cv. 'Sugraone' genome (kindly provided by the Computational Biology Unit at FEM). To confirm the integrity of the predicted off-targets, Sanger sequencing was performed on the DNA amplicons obtained from KO lines using the primers in Table S8.

***S1.3 Expression analysis of PME10 OE lines***

Total RNA was extracted from leaf tissue using the Spectrum™ Plant Total RNA Kit (Sigma-Aldrich), treated with DNaseI (TURBO DNA-free™ Kit, Invitrogen™) and the first-strand cDNA was synthesised using SuperScript™ III Reverse Transcriptase (Invitrogen™), using 2 µg of RNA according to the manufacturer's instructions. qPCR reactions were carried out using the kit qPCRBIO SyGreen® Mix Separate-ROX (PCR Biosystems) in a ViiA 7 Real-Time PCR System (Applied Biosystems). The *PME10* expression level was evaluated using specific primers for the gene, using *ACT* (*Vitvi04g01613*) and *GAPDH (Vitvi17g01598*) as normaliser genes (Table S8). The specificity of the primers was also checked by means of melting curve analysis (Figure S10). Briefly, each reaction was carried out in 12.5 µL and consisted of 1X qPCRBIO SyGreen Mix, 0.4 µM primers, using 10 ng of cDNA as template. Raw data was analysed using LinRegPCR (Ruijter *et al.*, 2009), and the expression was estimated as normalised relative quantity (NRQ) as reported by (Hellemans *et al.*, 2008).

***S1.4 T-DNA Copy Number analysis* of *VviPME10* OE lines**

The copies of integration of the T-DNA in the plant genome of *VviPME10* OE lines was evaluated through the nptII gene quantification using a C1000 thermal cycler and a CFX96 real-time PCR system. The real-time PCR reaction was performed in a final volume of 12.5 μl containing 40 ng genomic DNA and 0.4 μM primers. The thermal protocol was as follows: polymerase activation for 30 s at 95 °C, 40 cycles of denaturation for 5 s at 95 °C and annealing/extension for 15 s at 58 °C, followed by a melting curve protocol: 95 °C for 10 s, 65 °C for 5 s, then a stepwise T increase (0.5 °C/s) up to 95 °C with continuous detection. The primers used to amplify the endogenous grapevine gene *VviCHI* and the exogenous gene *nptII* are shown in Table S8. The standard curves (four points, starting from 10^6^ plasmid molecules and using a serial dilution of 1:5) were generated using a plasmid pGEM-T easy (Promega, Madison, Wisconsin, USA) containing specific *VviCHI* and *nptII* gene fragments. For each line, the transgene CN was calculated using the formula: (total transgene copies/total endogenous gene copies) × 2. Total copies of the transgene and the endogenous gene were obtained from the standard curves based on the mean values of the quantification cycles (Cq).

***S1.5 Acclimation and growth characterisation of OE and KO lines***

Once rooted, three biological replicates of healthy transgenic and control grapevine plants were acclimated in the greenhouse. The plants were transferred to 400 mL square plastic pots filled with sterilised growing medium (TerComposti, Italy). Acclimation was carried out under controlled light conditions (~100 μmol m-2·s-1 PPFD, 24°C and a 16/8 h light/dark photoperiod). The humidity was gradually reduced. The plants were transferred to 750 mL pots containing the same substrate and transferred to the greenhouse. The leaf area (cm2) of mature leaves from three replicates of control and transgenic plants was estimated. The lengths of the central veins of the leaves (cm) were measured and the data were used to calculate the leaf area using the formula (0,9306 *(POWER(central vein lenght;1,9558)). Internode length (cm) was measured by averaging the length of 5 consecutive internodes on the main stem per plant. Total height (cm) was determined using a meter.

**Methods S2. Detailed procedures for Bc artificial inoculation assays.**

***S2.1 Botrytis cinerea artificial inoculation of flowers of different grapevine genotypes***

Inflorescences at full cap-fall stage [EL25/26, according to (Eichhorn and Lorenz, 1977)] were sprayed with the *Bc* suspension and with media without conidia (mock inoculation) until fully covered. Then, the inflorescences were bagged in clear plastic bags, sprayed with 50 mL of water, for 24 hours (h) to ensure high humidity, essential for conidial germination, according to (Haile *et al.*, 2017). After 24 h, the bags were removed, and three to four inflorescences from treated and control plants were collected at 24 h post inoculation (hpi) (first experiment), and at 24 and 96 hpi (second experiment), and snap-frozen until use. In the second experiment, berry samples were also collected (two clusters/replicate), from treated and control plants, at 12 weeks post inoculation (wpi) as described by (Haile *et al.*, 2020). The experimental procedure is summarised in Figure S1.

To confirm *Bc* infection of grapevine flowers, genomic DNA was extracted from treated samples following the method by (Allen *et al.*, 2006). The quantification of DNA of *Bc* in the presence of DNA of *V. vinifera* was estimated following the method from (Ammour *et al.*, 2019) using a qPCR assay and was expressed in terms of a colonization coefficient (CC), which was the ratio between DNA amounts of *Bc* and *V. vinifera*. After extraction, 20 ng of DNA from each sample was used for testing. The qPCR assay was based on two primers, specific for *Bc3* (*AM233400.1*) and *VviGAPDH (Vitvi17g01598)* (Table S8), to quantify *Bc* and *Vv* DNA, respectively. Reaction was performed using the KAPA SYBR® FAST qPCR Kit (Merck) on a ViiA 7 Real-Time PCR System (Applied Biosystems), using an initial denaturation at 95°C for 3 min, followed by 40 cycles and a melting curve. Three controls were used: DNA from *Bc* mycelium, DNA from *V. vinifera* flowers and DNA from berries infected with *Bc*. The melting curves of infected and control samples were compared with the PCR controls. Data was analysed using LinRegPCR (Ruijter *et al.*, 2009) to estimate the amount of DNA from *Bc* and *Vv* in each sample. A DNA standard curve was constructed for *Bc3* and *VviGAPDH* amplicons.

***S2.2 Botrytis cinerea artificial leaf inoculation assays of KO and OE lines***

Leaves from the *VviPME10* KO and OE lines, as well as the control plants, were inoculated using the protocol by (Vega *et al.*, 2015). Three healthy, developed leaves were selected from each plant. The leaves were surface-sterilised with 1% Sodium hypochlorite solution for 10 minutes and washed four times with distilled water. After sterilisation, the leaves were dried and inoculated with the *Bc* suspension, leaving three spots (1500 conidia each) from one side of the leaf midrib and a control without conidia from the other side. Inoculated leaves were placed in sealed boxes over sterile paper soaked with sterile distilled water to increase the humidity. The boxes were transferred to the growth chamber at 24°C, 16/8 light/dark. *Bc* infection was evaluated at 5 days post inoculation (dpi) in terms of disease severity, calculated as the average leaf area covered with brown necrotic symptoms (mm^2^). The lesion area (mm^2^) of the infected region of each leaf was measured with Image J (Abramoff *et al.*, 2004).

**Methods S3. Detailed experimental procedures for biochemical and immunohistochemical analyses.**

***S3.1 Determination of methyl ester content and monosaccharide composition in cell wall of flower and berry skin samples***

Alcohol-Insoluble Residue (AIR) extraction was performed as previously described (Lionetti *et al.*, 2017). The degree of methyl esterification in CW of *B. cinerea*-infected and mock-inoculated flower and berry skin samples was assessed using a microplate-adapted alcohol oxidase/acetylacetone method (Klavons and Bennett, 1986). De-starched AIR (1 mg) was suspended in 30 μL water and 10 μL of 1 M NaOH, incubated for 1 h at room temperature, and neutralised with 10 μL 1 M HCl. After centrifugation (14,000×g, 5 min), 4 μL supernatant was loaded into 96-well plates (Costar) and diluted to 50 μL. Alcohol oxidase (50 μL; 0.03 units in 0.1 M sodium phosphate, pH 7.0) was added, and the mixture was incubated for 15 min at room temperature. Then, 100 μL of a 2,4-pentanedione/ammonium acetate/acetic acid solution was added, followed by a 10-min incubation at 68°C. Samples were cooled, and absorbance was measured at 412 nm. Methanol content was calculated as previously described (Klavons and Bennett, 1986). For CW monosaccharide composition, 2 mg of de-starched AIR was hydrolyzed in 200 μL of 2 M TFA at 121°C for 90 min. Post-hydrolysis, 200 μL isopropanol was added, and samples evaporated at 40°C incubated in 200 µL of 2 M TFA at 121°C. After 90 min, 200 µL of isopropanol was added, and the mixtures were evaporated at 40°C with a stream of N_2_ gas. The TFA-hydrolyzed monosaccharides were suspended in 200 µL of water. The monosaccharide composition of de-starched and TFA-hydrolyzed AIR was determined by high-performance anion-exchange chromatography with pulsed amperometric detection using a PA20 column (Dionex). Peaks were identified and quantified by comparison with a standard mixture of fucose, rhamnose, arabinose, galactose, glucose, xylose, mannose, galacturonic acid and glucuronic acid (Sigma-Aldrich).

***S3.2 PME Activity Determination***

Protein extracts were prepared by homogenising uninfected and infected grapevine tissues in a buffer containing 1 M NaCl, 12.5 mM citric acid, 50 mM Na2HPO4, 1% polyvinylpyrrolidone, 0.02% sodium azide, and protease inhibitor (1:100 v/v), pH 7.0. After shaking (3 h, 4°C) and centrifugation (15,000×g, 15 min), protein concentrations in supernatants were measured by the Bradford assay using BSA as a standard. PME activity was evaluated using the PECTOPLATE assay (Lionetti, 2015). Square Petri dishes (Greiner Bio-One) were prepared with 0.1% (w/v) of apple pectin (Sigma-Aldrich), 1% (w/v) SeaKem® LE agarose (Lonza Bioscience), 12.5 mM citric acid and 50 mM Na_2_HPO_4_, pH 7.0. Equal amounts of protein (1 µg in 20 μL) were loaded into each well. Plates were incubated at 30°C for 16 h, then stained with 0.05% Ruthenium Red for 30 min. Fuchsia-stained halos, indicative of pectin de-methyl esterification, were measured using ImageJ (Abramoff *et al.*, 2004). PME activity was calculated using a standard curve generated with commercial PME (Sigma-Aldrich).

***S3.3 Immunohistochemical analysis***

Samples for immunohistochemistry were immersed in FAA solution and vacuumed for 10 min. Following fixation, the tissue was dehydrated in ethanol using increasing concentrations and embedded in Paraplast Plus (Sigma-Aldrich) as described in (Rojas *et al.*, 2021). Paraplast was removed from 5 µm sections using 100% xylol and chloroform for 60 and 30 min respectively; and rehydrated in decreasing concentrations of ethanol in PBS pH 7.4, (100, 70, 50 and 30% ethanol) with 5 minute incubations. Finally, sections were shaker incubated for 60 minutes in PBS. Prior to immunolabeling, rehydrated sections were blocked with 5% skimmed milk and 0.4% Triton X-100, and washed three times in PBS. Primary antibody incubation took place during 16 h. using LM19 or LM20 (1:10 in 5% skimmed milk) as primary antibodies (rat monoclonal IgM, Verhertbruggen et al., 2009), which bind to de-methylesterified and methylesterified pectin respectively. After three 5 min PBS washing steps, sections were incubated with a 1:300 dilution of Goat anti-rat IgG (H+L) Highly Cross-Adsorbed Secondary Antibody, Alexa Fluor™ Plus 488; for 2 h at 4ºC. After secondary antibody removal, samples were washed in PBS four times and incubated with Calcofluor White M2R in PBS (0.25 g L^-1^), dried, mounted using Neo-mount and kept at 4ºC. Confocal microscopy was performed on a TCS SP8 Leica confocal microscope. exciting the samples with a 405 nm Diode and 488 nm OPSL laser. Fluorescence detection was done using two PMT detectors for wavelengths between 410 and 493 nm for Calcofluor White, and 493 to 540 nm for the secondary antibody. Mean fluorescence intensity was measured using the Leica LASX software, with three measure**ments per tissue from three independent flowers of each cultivar.**

**Methods S4. Computational and gene expression analysis workflows.**

***S4.1 VviPME* gene family identification: manual curation and phylogenetic analysis**

The V*viPME* gene family was identified using HMMER (Finn *et al.*, 2011) with default settings against the Pfam database (PfamA, release 36.0). The VCost.v3 and V4 annotations of the 12X.v2 (Canaguier *et al.*, 2017) and PN40024.v4 (Velt *et al.*, 2023) reference genome assemblies were analysed for the presence of the PME domain (Pfam01095), common to both group I and group II PMEs, and of the PRO region (Pfam04043), exclusive to members of group II PMEs (Pelloux *et al.*, 2007). Results were filtered using a domain E value cut-off of ≤1×10^-5.^

For phylogenetic analysis, grapevine protein sequences (VviPME) were aligned to *A. thaliana* sequences (AtPME) (Louvet *et al.*, 2006) using MAFFT with default parameters (Katoh & Standley, 2013). A maximum likelihood tree was constructed using IQ-TREE supported by 1000 bootstrap replicates (Hoang *et al.*, 2018; Nguyen *et al.*, 2015) and visualised using Figtree (<http://tree.bio.ed.ac.uk/software/figtree/>).

***S4. 2 Gene expression analyses***

Total RNA was extracted from flowers and berry skins using the Spectrum™ Plant Total RNA Kit (Sigma-Aldrich) and treated with DNase I using the TURBO DNA-free™ Kit (Invitrogen™), following the manufacturers’ instructions.

*RNA sequencing, data processing and data analysis*

Illumina mRNA sequencing libraries (non-stranded, poly(A)-enriched) were prepared by Novogene (UK) using the Novogene NGS RNA Library Prep Set (PT042) * based on the NEB Next® Ultra™ RNA Library Prep Kit and NEB Next® and sequenced on the NovaSeq 6000 Illumina platform. All raw RNA-Seq read data were deposited in the NCBI Short Read Archive (<http://www.ncbi.nlm.nih.gov/sra/>) under the BioProject accession code PRJNA1031966. Illumina reads were pre-processed, as described above, and then mapped and counted using STAR (Dobin *et al.*, 2013) and featureCounts (Liao *et al.*, 2014) to a concatenated version of the *V. vinifera* PN40024 12x.v2 genome assembly (Canaguier *et al.*, 2017) and *B. cinerea* DW1 genome assembly (Blanco-Ulate *et al.*, 2013). The summary of the results of the previous steps are presented in Table S2. Genes were filtered by expression using the filterByExpr function of edgeR R package (Robinson et al., 2010). Differential expression analysis was performed using the R package limma (Ritchie et al., 2015). The complete list of differentially expressed genes (DEGs) is in Table S3. MapMan enrichment analyses of DEGs were conducted using the gprofiler2 R package (Kolberg *et al*., 2020) with default parameters. Statistical significance was assessed using a Benjamini–Hochberg adjusted P-value threshold of 0.05 (Benjamini and Hochberg, 1995).

*Quantitative polymerase chain reaction analysis*

The first-strand cDNA was synthesised using SuperScript™ III Reverse Transcriptase (Invitrogen™), using 2 µg of DNAseI-treated RNA according to the manufacturer's instructions. qPCR reactions were carried out using the kit qPCRBIO SyGreen® Mix Separate-ROX (PCR Biosystems) in a ViiA 7 Real-Time PCR System (Applied Biosystems). Expression level was evaluated using specific primers for the targeted genes, and using *ACT* (*Vitvi04g01613*), *ATP16* (*Vitvi03g00055*), *UBIQ* (*Vitvi19g00434*) and *EF1α* (*Vitvi06g00319*) as normaliser genes (Table S8). The specificity of the primers was also checked using melting curve analysis performed during qPCR reactions (Figure S10). Briefly, each reaction was carried out in 12.5 µL and consisted of 1X qPCRBIO SyGreen Mix, 0.4 µM primers, using 10 ng of cDNA as template. Raw data was analysed using LinRegPCR (Ruijter *et al.*, 2009), and the expression was estimated as Normalised Relative Quantity (NRQ) as reported by (Hellemans *et al.*, 2008).

***S4.3 Implementation of the Botrytis Stress Atlas Explorer***

A plant-pathogen gene expression atlas explorer was constructed with a specific focus on *Bc*-grapevine interaction. The NCBI Sequence Read Archive (SRA) and Gene Expression Omnibus (GEO) databases were searched for transcriptome (RNAseq) experiments related to *Bc*-grapevine berry interaction. The resulting experiments were downloaded in SRA format and converted to fastq using fastq-dump. Illumina reads from our previously conducted RNA-seq experiment in flowers were also included in the analysis. All the Illumina reads were trimmed using Fastp software (Chen *et al.*, 2018) followed by STAR mapping (Dobin *et al.*, 2013) to a concatenated version of the *V. vinifera* PN40024 (12x.v2 assembly) (Canaguier *et al.*, 2017) and *B. cinerea* DW1 genome assembly (Blanco-Ulate *et al.*, 2013). Raw counts were calculated using Feature Counts (Liao *et al.*, 2014), with only reads mapping to the grapevine genome used in subsequent analyses. Genes were filtered by expression using the filterByExpr function of edgeR R package (Robinson et al., 2010). Differential expression analysis was performed as described above. The results of the analysis were then made available as an app on the Vitviz suite within the PlantaeViz platform (<http://plantaeviz.tomsbiolab.com/vitviz/botrytis_atlas/>), showing a heatmap with the log2FC for each comparison of the different experiments as the colour of the cell and the corresponding -log (p-value) as the size of the dot in each cell.

**Methods S5. Detailed experimental procedures for DAP-seq and DAP-qPCR analyses, PME10 promoter analysis and cloning, and dual luciferase assay.**

***S5.1 DAP-seq and DAP-qPCR analyses***

Public WRKY03 DAP-seq data was downloaded from SRA (PRJNA1199911; GST-Halo tag) and analyzed as in (Zhang *et al.*, 2023). Sequencing reads were mapped to the PN40024 (12X.2) genome assembly available at GRAPEDIA (https://grapedia.org/). Enrichment of binding events was first evaluated by plotting genome coverage compared with the negative control (Bartlett et al. 2017). To identify “peaks,” enriched regions relative to the control that correspond to binding events, the GEM peak caller was used (Guo *et al*., 2012), which performs simultaneous peak calling and motif refinement (q=2). *De novo* motif discovery was performed using 200 bp sequences centered at GEM-identified binding events for the 600 most enriched peaks (Bailey *et al.*, 2009). WRKY03 target genes were identified by associating the peak regions to the TSS of the closest genes. For DAP-qPCR analysis, new TF (n=3) and Input (n=3) libraries were generated. WRKY03 was synthesized (GeneArt, Thermo Fisher Scientific) based on the PN genome, and cloned into the pIX-HALO expression plasmid (TAIR Vector:6530264275) to create an expression vector that contained a HaloTag in frame at the N-terminus. gDNA was purified from cv. ‘Tempranillo’ young leaves, and gDNA library and DAP-libraries were generated following the published protocol of (Bartlett *et al.*, 2017). Briefly, the gDNA sample was sheared into ~200 bp fragments using a Covaris Focus-ultrasonicator and subjected to end repair, A-tailing, and adapter ligation to generate Illumina-compatible libraries. HALO-tagged WRKY03 protein was synthesized in vitro using the TNT® Coupled Reticulocyte Lysate System (Promega) and captured with Magne® HaloTag® Beads (Promega). The gDNA libraries were incubated with the protein-bead complexes for DNA affinity purification (DAP). Bound DNA was eluted, PCR-amplified, and 12 ng of purified DNA from each library was used for qPCR with the NZYSpeedy qPCR Green Master Mix (NZYTech). qPCR conditions, primers and TM are detailed in Table S7.

***S5.2 PME10 promoter sequencing and WRKY03 binding sites identification***

Genomic DNA was extracted from leaves obtained from plants of the reference genotype PN40024 following the CTAB DNA extraction protocol (Allen *et al.*, 2006). The *PME10* (*Vitvi06g01152*) promoter (2 kb upstream of the *PME10* CDS start codon in the PN40024 gene prediction) was amplified using Phusion Hot Start II DNA Polymerase (Thermo Scientific™) and the primers in Table S8 and sequenced by Sanger sequencing. Putative WRKY03 binding sites were identified based on the 21-nt WRKY03 letter-probability matrix (with the core sequence TTGAC), derived from the 600 peaks with the highest scores in the mapping the WRKY03 DAP-seq data onto the PN40024 reference genome (personal communication; PRJNA1199911).

The letter-probability matrix of WRKY03, reported in Data S1, was used to screen the *PME10* promoter sequence retrieved from the PN40024 assembly (up to 2kb from ATG) using FIMO (Find Individual Motives Occurrences) tool from the MEME suite (Grant *et al*., 2011) with a *p-*value threshold=10^-4^.

***S5.3 Dual luciferase assay***

The amplified *VviPME10-*PN40024 promoter was cloned into the pPGWL7.0 reporter vector(Karimi *et al.*, 2002), using Gateway™ LR Clonase™ II Enzyme Mix (Invitrogen™, USA) according to the manufacturer's instructions, to control the firefly luciferase gene (LUC). The pPGWL7-*VviPME10* pro:LUC reporter vector, the pK7WG2 -CaMV35S-P:*VviWRKY03* effector vector, and the *Renilla reniformis* (REN) reference vector (pK7WG2 - CaMV35S-P: REN) were transferred to *Rhizobium radiobacter* (*A. tumefaciens)* strain C58C1-pGV2260 by electroporation. Agrobacterium harboring the vector pCH32-35S:p19 which expresses the silencing suppressor p19 of tomato bushy stunt virus was also used. Bacterias were cultured in liquid media to late exponential phase and cells were harvested by centrifugation at 3000 g for 15 minutes at room temperature (24 ºC). Cell pellets were resuspended in agroinfiltration buffer (10 mM morpholinoethanesulphonic “MES” acid-KOH, 10 mM MgCl2 and 150 mM acetosyringone; pH 5.6) and incubated for 3 h at room temperature (24 ºC). These cells were mixed in 1:1 ratio (final OD600 of 0.2 for each plasmid), and then injected into young fully expanded leaves of 4-week-old *Nicotiana benthamiana* plants (Schöb *et al.*, 1997). Dual Luciferase Assay was carried out as described by (D’Incà *et al.*, 2023), using the Dual-Luciferase® Reporter (DLR™) Assay System (Promega). Results were obtained from 4-5 independent plants transformed in 3 different leaves. Three days after infiltration, 3 disks per leaf were quantified. In total, we got 27 data points for control condition and 17 data points for the effector. *Firefly* and *R. reniformis* luminescence were detected using a Tecan Infinite M200 PLEX instrument and the data are presented as the ratio of the two measurements relativized to control condition

Abramoff, M., Magalhães, P., and Ram, S.J. (2004) Image Processing with ImageJ. *Biophotonics Int.*, **11**, 36–42.

Allen, G.C., Flores-Vergara, M.A., Krasnyanski, S., Kumar, S., and Thompson, W.F. (2006) A modified protocol for rapid DNA isolation from plant tissues using cetyltrimethylammonium bromide. *Nat. Protoc.*, **1**, 2320–2325.

Ammour, M.S., Fedele, G., Morcia, C., Terzi, V., and Rossi, V. (2019) Quantification of botrytis cinerea in grapevine bunch trash by real-time PCR. *Phytopathology*, **109**, 1312–1319.

Bailey, T.L., Boden, M., Buske, F.A., Frith, M., Grant, C.E., Clementi, L., et al. (2009) MEME Suite: tools for motif discovery and searching. *Nucleic Acids Res*., **37** (Web Server issue), W202-8.

Bartlett, A., O’Malley, R.C., Huang, S.S.C., Galli, M., Nery, J.R., Gallavotti, A, et al. (2007) Mapping genome-wide transcription-factor binding sites using DAP-seq. *Nat Protoc*.**12**(8), 1659–1672.

Benjamini, Y. and Hochberg, Y. (1995) Controlling the false discovery rate: a practical and powerful approach to multiple testing. *Journal of the Royal Statistical Society: Series B: Methodological*, **57**, 289–300.

Blanco-Ulate, B., Allen, G., Powell, A.L.T., and Cantu, D. (2013) Draft genome sequence of Botrytis cinerea BcDW1, inoculum for noble rot of grape berries. *Genome Announc.*, **1**, 13–14.

Canaguier, A., Grimplet, J., Di Gaspero, G., Scalabrin, S., Duchêne, E., Choisne, N., et al. (2017) A new version of the grapevine reference genome assembly (12X.v2) and of its annotation (VCost.v3). *Genomics data*, **14**, 56–62.

Chen, S., Zhou, Y., Chen, Y., and Gu, J. (2018) Fastp: An ultra-fast all-in-one FASTQ preprocessor. *Bioinformatics*, **34**, i884–i890.

Clement, K., Rees, H., Canver, M.C., Gehrke, J.M., Farouni, R., Hsu, J.Y., et al. (2019) CRISPResso2 provides accurate and rapid genome editing sequence analysis. *Nat. Biotechnol.*, **37**, 224–226.

Concordet, J.P. and Haeussler, M. (2018) CRISPOR: Intuitive guide selection for CRISPR/Cas9 genome editing experiments and screens. *Nucleic Acids Res.*, **46**, W242–W245.

D’Incà, E., Foresti, C., Orduña, L., Amato, A., Vandelle, E., Santiago, A., et al. (2023) The transcription factor VviNAC60 regulates senescence- and ripening-related processes in grapevine. *Plant Physiol.*, **192**, 1928–1946.

Dobin, A., Davis, C.A., Schlesinger, F., Drenkow, J., Zaleski, C., Jha, S., et al. (2013) STAR: Ultrafast universal RNA-seq aligner. *Bioinformatics*, **29**, 15–21.

Eichhorn, K.W. and Lorenz, H. (1977) Phaenologische Entwicklungstadien der Rebe. *Nachrichtenblatt des Dtsch. Pflanzenschutzdienstes*, **29**, 119–120.

Finn, R.D., Clements, J., and Eddy, S.R. (2011) HMMER web server: interactive sequence similarity searching. *Nucleic Acids Res.*, **39**, W29–W37.

Grant, C.E., Bailey, T.L., Noble, W.S. (2011) FIMO: scanning for occurrences of a given motif. *Bioinformatics*, **27**(7):1017-8.

Guo, Y, Mahony, S., Gifford, D.K. High resolution genome wide binding event finding and motif discovery reveals transcription factor spatial binding constraints (2012) *PLoS Comput Biol*. **8**(8):e1002638.

Haile, Z.M., Malacarne, G., Pilati, S., Sonego, P., Moretto, M., Masuero, D., et al. (2020) Dual Transcriptome and Metabolic Analysis of *Vitis vinifera* cv. Pinot Noir Berry and *Botrytis cinerea* During Quiescence and Egressed Infection. *Front. Plant Sci.*, **10**, 1704.

Haile, Z.M., Pilati, S., Sonego, P., Malacarne, G., Vrhovsek, U., Engelen, K., et al. (2017) Molecular analysis of the early interaction between the grapevine flower and *Botrytis cinerea* reveals that prompt activation of specific host pathways leads to fungus quiescence. *Plant Cell Environ.*, **40**, 1409–1428.

Hellemans, J., Mortier, G., De Paepe, A., Speleman, F., and Vandesompele, J. (2008) qBase relative quantification framework and software for management and automated analysis of real-time quantitative PCR data. *Genome Biol.*, **8**, R19.

Hoang, D.T., Chernomor, O., Von Haeseler, A., Minh, B.Q., and Vinh, L.S. (2018) UFBoot2: Improving the ultrafast bootstrap approximation. *Mol. Biol. Evol.*, **35**, 518–522.

Karimi, M., Inzé, D., and Depicker, A. (2002) GATEWAY^TM^ vectors for Agrobacterium-mediated plant transformation. *Trends Plant Sci.*, **7**, 193–195.

Klavons, J.A. and Bennett, R.D. (1986) Determination of Methanol Using Alcohol Oxidase and Its Application to Methyl Ester Content of Pectins. *J. Agric. Food Chem.*, **34**, 597–599.

Kolberg, L., Raudvere, U., Kuzmin, I., Vilo, J., and Peterson, H. (2020) gprofiler2 – an R package for gene list functional enrichment analysis and namespace conversion toolset g:Profiler. *F1000Research*, **9**, 709.

Liao, Y., Smyth, G.K., and Shi, W. (2014) FeatureCounts: An efficient general purpose program for assigning sequence reads to genomic features. *Bioinformatics*, **30**, 923–930.

Lionetti, V. (2015) PECTOPLATE: the simultaneous phenotyping of pectin methylesterases, pectinases, and oligogalacturonides in plants during biotic stresses. *Front. Plant Sci*, **6**, 331.

Lionetti, V., Fabri, E., De Caroli, M., Hansen, A.R., Willats, W.G.T., Piro, G., and Bellincampi, D. (2017) Three pectin methylesterase inhibitors protect cell wall integrity for arabidopsis immunity to Botrytis. *Plant Physiol.*, **173**, 1844–1863.

Louvet, R., Cavel, E., Gutierrez, L., Guénin, S., Roger, D., Gillet, F., et al. (2006) Comprehensive expression profiling of the pectin methylesterase gene family during silique development in Arabidopsis thaliana. *Planta*, **224**, 782–791.

Love, M.I., Huber, W., and Anders, S. (2014) Moderated estimation of fold change and dispersion for RNA-seq data with DESeq2. *Genome Biol.*, **15**, 1–21.

Nguyen, L.T., Schmidt, H.A., Von Haeseler, A., and Minh, B.Q. (2015) IQ-TREE: A fast and effective stochastic algorithm for estimating maximum-likelihood phylogenies. *Mol. Biol. Evol.*, **32**, 268–274.

Pelloux, J., Rustérucci, C., and Mellerowicz, E.J. (2007) New insights into pectin methylesterase structure and function. *Trends Plant Sci.*, **12**, 267–277.

Ritchie, M.E., Phipson, B., Wu, D., Hu, Y., Law, C.W., Shi, W., et al. (2015) Limma powers differential expression analyses for RNA-sequencing and microarray studies. *Nucleic Acids Res*. **43**(7):e47.

Robinson, M.D., McCarthy D.J., Smyth G.K. (2010) edgeR: a Bioconductor package for differential expression analysis of digital gene expression data. *Bioinformatics*. **26**(1), 139-140.

Rojas, B., Suárez-Vega, F., Saez-Aguayo, S., Olmedo, P., Zepeda, B., Delgado-Rioseco, J., et al. (2021) Pre-anthesis cytokinin applications increase table grape berry firmness by modulating cell wall polysaccharides. *Plants*, **10**, 2642.

Ruijter, J.M., Ramakers, C., Hoogaars, W.M.H., Karlen, Y., Bakker, O., van den hoff, M.J.B., and Moorman, A.F.M. (2009) Amplification efficiency: Linking baseline and bias in the analysis of quantitative PCR data. *Nucleic Acids Res.*, **37**, e45.

Schöb, H., Kunz, C., and Meins, F. (1997) Silencing of transgenes introduced into leaves by agroinfiltration: A simple, rapid method for investigating sequence requirements for gene silencing. *Mol. Gen. Genet.*, **256**, 581–585.

Vega, A., Canessa, P., Hoppe, G., Retamal, I., Moyano, T.C., Canales, J., et al. (2015) Transcriptome analysis reveals regulatory networks underlying differential susceptibility to Botrytis cinerea in response to nitrogen availability in Solanum lycopersicum. *Front. Plant Sci.*, **6**, 163341.

Velt, A., Frommer, B., Blanc, S., Holtgräwe, D., Duchêne, É., Dumas, V., et al. (2023) An improved reference of the grapevine genome reasserts the origin of the PN40024 highly homozygous genotype. *G3 Genes|Genomes|Genetics*, **13**, 67.

Zhang C., Dai Z., Ferrier T., Orduña L., Santiago A., Peris A., et al. (2023) MYB24 orchestrates terpene and flavonol metabolism as light responses to anthocyanin depletion in variegated grape berries. *Plant Cell*, **35**(12):4238-4265.
