# Supplementary material for: PME10 Is a Pectin Methylesterase Driving PME Activity and Immunity Against Botrytis cinerea in Grapevine (Vitis vinifera L.)
Source: Plant Biotechnol J. 2025 Jul 29;23(11):4981–97. doi: 10.1111/pbi.70279 (PMC12576464; doi:10.1111/pbi.70279)
Supplement: Supplementary file 2 — Figure S1. Monosaccharide compositions of cell wall extracts from flowers and berry skins of different grapevine genotypes. Figure S2. Phylogenetic tree of Arabidopsis thaliana and Vitis vinifera Pectin Methyl Esterase (PME) genes. Figure S3. Expression profiles of PME family genes across various grapevine organs and tissues at different developmental stages. Figure S4. Summary of RNA‐seq results comparing Bc‐infected and control flowers of ‘Souvigner Gris’ (SG) and ‘Teroldego’ (TE) at 24 h post‐inoculation. Figure S5. Summary of on‐target analysis of the PME10 knockout (KO) lines. Figure S6. Summary of the off‐target analysis of the PME10 KO lines. Figure S7. Phenotypic characterisation of PME10 KO lines compared with control plants. Figure S8. Phenotypic characterisation of PME10 overexpressing (OE) lines compared with control plants. Figure S9. WRKY03 DAP‐seq and DAP‐qPCR analyses of the WRKY03‐PME10 interaction. Figure S10. Melting curve analysis during qPCR assays using primers for PME10, PME11, and PME12. Table S1. Complete list of the 62 PME genes identified in the V. vinifera PN40024 reference genome. Table S2. Summary of the Illumina read processing and mapping to the concatenated V. vinifera PN40024 12X.v2 and B. cinerea DW1 genome assemblies. Table S3. Differentially expressed genes in ‘Souvigner Gris’ and V. vinifera ‘Teroldego’ flowers at 24 h post‐inoculation with B. cinerea. Table S4. Metadata of publicly available RNA‐seq experiments on B. cinerea ‐grapevine berry interactions, included in the Botrytis Stress Atlas Explorer. Table S5. Predicted PME10 off‐target regions in V. vinifera ‘PN40024’ and ‘Sugraone’ genome assemblies. Table S6. WRKY03‐binding events on PME genes detected by DAP‐seq analysis. Table S7. PME10 DAP‐seq qPCR conditions. Table S8. List of primers used throughout the study. Methods S1. Molecular analysis and acclimation procedures for PME10 OE and KO lines. Methods S2. Detailed procedures for Bc artificial inoculation assays. Metho [file PBI-23-4981-s002.zip › pbi70279-sup-0014-Caption.docx]

**Figure S1.** **Monosaccharide composition of cell wall extracts from flowers and berry skins of different grapevine genotypes.**

Relative abundance of arabinose (Ara), fucose (Fuc), galactose (Gal), galacturonic acid (Gal A), glucose (Glu), glucuronic acid (Glc A), mannose (Man), rhamnose (Rha), and xylose (Xyl) in protein extracts derived from uninfected and *B. cinerea*-infected samples. The relative abundance of each monosaccharide is expressed as a molar percentage, calculated by dividing the number of moles of each sugar by the total moles of all detected monosaccharides. (a) Flowers of ‘Souvignier Gris’ (SG) and cv. ‘Teroldego’ (TE) collected at 24 hours post-inoculation (hpi). (b) Flowers of SG and cv. ‘Sangiovese’ (SN) collected at 24 and 96 hpi. (c) Berry skins of SG and SN collected at 12 weeks post-inoculation (wpi). Data are shown as mean ± SD (n = 3). No statistically significant differences were detected among treatments according to one-way ANOVA followed by Tukey’s post hoc test (P < 0.05). Ctrl = mock-inoculated control; Bc = *B. cinerea*-inoculated.

**Figure S2. Phylogenetic tree of *Arabidopsis thaliana* and *Vitis vinifera* pectin methyl esterase (PME) genes.**

Phylogenetic relationships between the *Arabidopsis thaliana* (*At*) and *Vitis vinifera* (*Vvi*) *PME* gene family members. The enlarged box shows in detail *PME* genes from clade 5A in close proximity to *AtPME17*, which has been shown to play a role in the resistance response to *Bc* in Arabidopsis (Del Corpo et al., 2020). The complete *VviPME* protein sequences were aligned with the *A. thaliana* PME protein sequences (Louvet et al., 2006) using MAFFT with default parameters (Katoh & Standley, 2013). A Maximum Likelihood tree was constructed using IQ-TREE, supported by 1000 bootstrap replicates (Nguyen et al., 2015; Thi Hoang et al., 2017) and visualized using Figtree (Rambaut, 2010).

**Figure S3. Expression profiles of PME family genes across various grapevine organs and developmental stages.**

A gene expression heatmap of *Vitis vinifera* PME genes was generated using the Corvina Atlas Explorer (<http://plantaeviz.tomsbiolab.com/vitviz/corvina_atlas/>). Expression values are presented on a logarithmic scale and correspond to robust multi-array average (RMA) normalized values. PHW = postharvest withering.

**Figure S4. Summary of RNA-seq results comparing *B. cinerea*-infected and control flowers of ‘Souvignier Gris’ (SG) and ‘Teroldego’ (TE) at 24 hours post-inoculation.**

(a) Venn diagrams showing the total number of differentially expressed genes (DEGs), including both upregulated and downregulated genes, in SG and TE. DEGs were defined as those with |log₂FC| ≥ 1 and adjusted *p*-value ≤ 0.05. The full list of DEGs is provided in Table S3. (b) Comparison of expression levels in commonly upregulated genes in both genotypes (n = 654), marked with an asterisk in (a). Linear regression (blue) shows a tendency of higher expression levels in the susceptible genotype (TE). (c) Bubble plot showing enriched MapMan functional categories among DEGs. (d) Co-expression heatmap of Pathogenesis-Related (PR) genes and defense-associated transcription factors in infected versus control samples of SG and TE. (e) TPM values of *VviPME10* in the same samples.

**Figure S5. Summary of on-target analysis of PME10 knock-out (KO) lines.**

Summary of Illumina MiSeq amplicon sequencing results. Mutations in the cleavage target site (insertion, deletion, and substitutions) were analyzed using CRISPResso2, version 2.1.3 (Clement *et al.*, 2019) by aligning Illumina reads of the 23 regenerated *PME10* KO lines compared to the cv. ‘Sugraone’ WT plant.

**Figure S6. Summary of off-target analysis of PME10 KO lines.**

a) Schematic representation of the four potential off-targets in *PME10* mutants. *PME10* paralogues from Clade 5A, *VviPME8*, *VviPME9*, *VviPME11* and *VviPME54*, are all located in chromosome 6. b) Identification of the PAM site (violet bar) and seed regions (green bar) in the four off-targets and alignment of the sequences of the four off-targets from lines *pme10*_KO_07 and *pme10*_KO_14 to the WT cv. ‘Sugraone’ sequence.

**Figure S7. Phenotypic characterization of PME10 KO lines compared with control plants.**

Representative images of *pme10*-KO_07 and *pme10*-KO_14 and control plants grown in 750 mL square plastic pots filled with sterilized growing medium (TerComposti, Italy) and maintained in a greenhouse. Scale bar = 14 cm. b) Plots of three phenotypic parameters, height, internode length, and total leaf area, measured in the *pme10* mutants and control plants. KO=knock-out

**Figure S8. Phenotypic characterization of PME10 overexpressing (OE) lines compared with control plants.**

Representative images of *PME10* OE-11 and *PME10* OE-22 and control plants grown in 750 mL square plastic pots filled with sterilized growing medium (TerComposti, Italy) and maintained in a greenhouse. Scale bar = 14 cm. b) Normalized relative quantity of *PME10* transcripts in OE lines and control plants. c) Plots of three phenotypic parameters, height, internode length, and total leaf area, measured in the overexpressing (OE) lines and control plants.

**Figure S9. WRKY03 DAP-seq and DAP-qPCR analyses of the WRKY03–PME10 interaction.**

(a) Schematic representation of three putative *WRKY03* binding sites identified within the 2000 bp region upstream of the *PME10* ATG start codon, which were selected for DAP-qPCR analysis. An additional upstream region (P1–Random), located ~2200 bp from the ATG, was included as a negative control. (b) DAP-qPCR results obtained using newly generated *WRKY03* and input DAP-seq libraries. Data are presented as mean ± SD (n = 3).

**Figure S10.** Melting curve analysis from qPCR assays using primers for *PME10*, *PME11*, and *PME12*.

**Table S1**. Complete list of the 62 *PME* genes identified in the *V. vinifera* PN40024 reference genome.

Each gene is identified by two different gene IDs inferred using the two different PN40024 genome assemblies (PN40024.v4.40X and 12X.v2) and annotations (V4 and VCost.v3) (Canaguier *et al*., 2017, Velt *et al*., 2023, respectively). To each gene, a name (gene symbol) was assigned based on a previous study (Khan *et al.,* 2019) and on the phylogenetic relationships obtained in this study (Figure S3). Each gene was also assigned to group I or II (based on the PME classification proposed by Pelloux et al. (2007), characterized by the presence of the PME domain (Pfam01095) or by the presence of both the PME domain (Pfam01095) and of the PRO region (Pfam04043).

**Table S2**. Summary of Illumina read processing and mapping to the concatenated *V. vinifera* PN40024 12x.v2 and *B. cinerea* DW1 genome assemblies.

A total of 12 RNAseq libraries were obtained from replicates of control and infected flowers of ‘Souvigner Gris’ and 'Teroldego' genotypes.

**Table S3.** Differentially expressed genes in ‘Souvigner Gris’ and *V. vinifera* ‘Teroldego’ flowers at 24 hours post-inoculation with *B. cinerea.*

Each gene is identified by the Gene ID and the gene description (PN40024 12X.v2 genome assembly, VCost.v3 genome annotation), and a Gene Symbol if the gene was present in the grapevine catalogue (catalogue v3 found at Grapedia; https://grapedia.org/genes/). To each gene, the Fold-change (FC) (Bc vs mock inoculated) and an adjusted p-value were assigned. The differentially expressed genes were those with log2FC ≥ 1 and log2FC ≤ -1, with an adjusted p-value ≤ 0.05.

**Table S4**. Metadata of publicly available RNAseq experiments on *B. cinerea*-grapevine berry interactions, included in the Botrytis Stress Atlas Explorer.

**Table S5.** Predicted *PME10* off-target regions in *V. vinifera* ‘PN40024’ and ‘Sugraone’ genome assemblies.

**Table S6.** WRKY03-binding events on *PME* genes detected by DAP-seq analysis.

**Table S7.** *PME10* DAP-seq qPCR conditions.

**Table S8.** List of primers used throughout the study.

**Data S1.** Letter-probability matrix for WRKY03 transcription factor binding sites (TFBS).

**Methods S1**. Molecular analysis and acclimation procedures for PME10 OE and KO lines.

**Methods S2.** Detailed procedures for Bc artificial inoculation assays.

**Methods S3.** Detailed experimental procedures for biochemical and immunohistochemical analyses.

**Methods S4.** Computational and gene expression analysis workflows.

**Methods S5.** Detailed experimental procedures for DAP-seq and DAP-qPCR analyses, *PME10* promoter analysis and cloning, and dual luciferase assay.
