# Supplementary figures and images for: PME10 Is a Pectin Methylesterase Driving PME Activity and Immunity Against Botrytis cinerea in Grapevine (Vitis vinifera L.)
Source: Plant Biotechnol J. 2025 Jul 29;23(11):4981–97. doi: 10.1111/pbi.70279 (PMC12576464; doi:10.1111/pbi.70279)

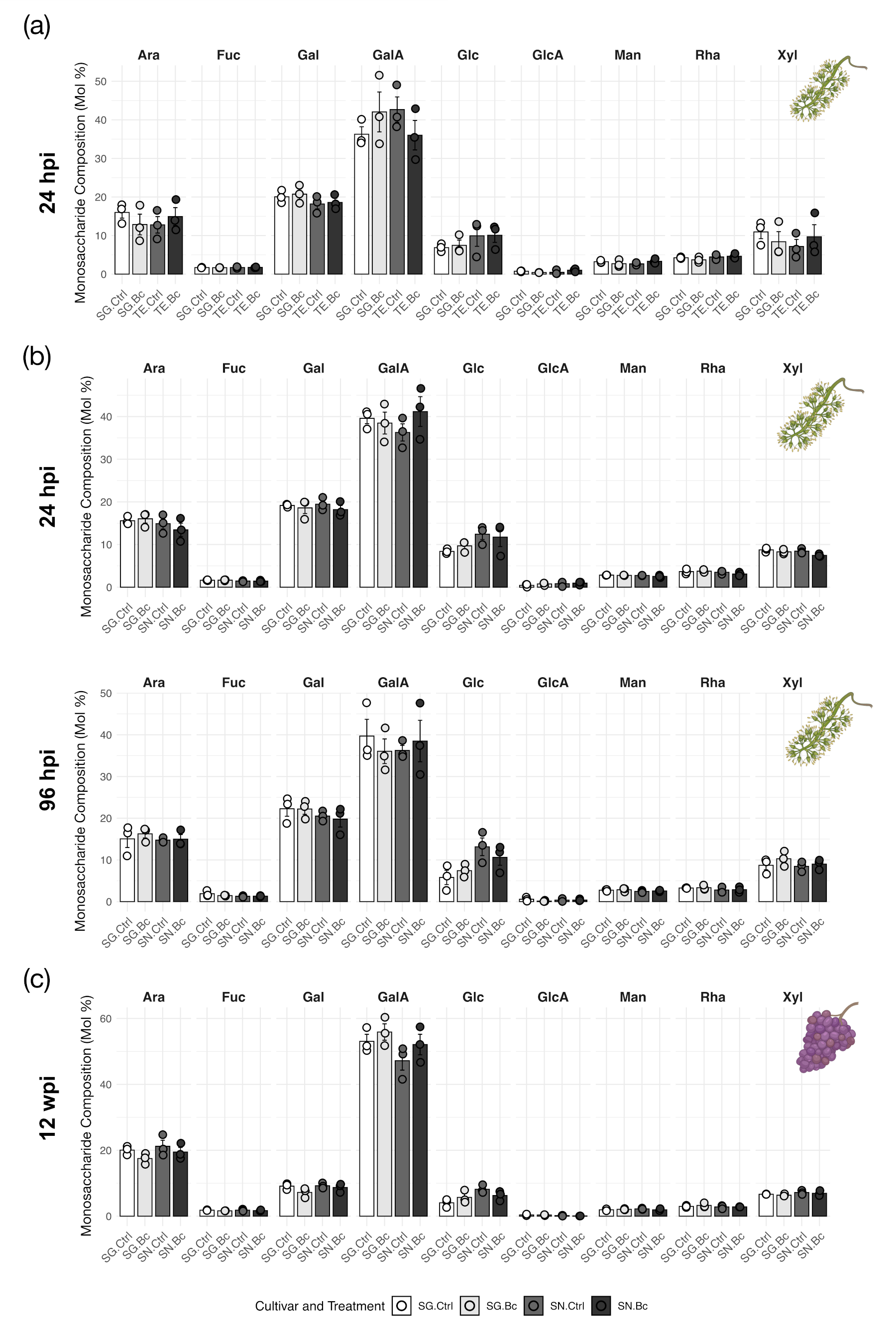

Supplement: Supplementary file 2 — Figure S1. Monosaccharide compositions of cell wall extracts from flowers and berry skins of different grapevine genotypes. Figure S2. Phylogenetic tree of Arabidopsis thaliana and Vitis vinifera Pectin Methyl Esterase (PME) genes. Figure S3. Expression profiles of PME family genes across various grapevine organs and tissues at different developmental stages. Figure S4. Summary of RNA‐seq results comparing Bc‐infected and control flowers of ‘Souvigner Gris’ (SG) and ‘Teroldego’ (TE) at 24 h post‐inoculation. Figure S5. Summary of on‐target analysis of the PME10 knockout (KO) lines. Figure S6. Summary of the off‐target analysis of the PME10 KO lines. Figure S7. Phenotypic characterisation of PME10 KO lines compared with control plants. Figure S8. Phenotypic characterisation of PME10 overexpressing (OE) lines compared with control plants. Figure S9. WRKY03 DAP‐seq and DAP‐qPCR analyses of the WRKY03‐PME10 interaction. Figure S10. Melting curve analysis during qPCR assays using primers for PME10, PME11, and PME12. Table S1. Complete list of the 62 PME genes identified in the V. vinifera PN40024 reference genome. Table S2. Summary of the Illumina read processing and mapping to the concatenated V. vinifera PN40024 12X.v2 and B. cinerea DW1 genome assemblies. Table S3. Differentially expressed genes in ‘Souvigner Gris’ and V. vinifera ‘Teroldego’ flowers at 24 h post‐inoculation with B. cinerea. Table S4. Metadata of publicly available RNA‐seq experiments on B. cinerea ‐grapevine berry interactions, included in the Botrytis Stress Atlas Explorer. Table S5. Predicted PME10 off‐target regions in V. vinifera ‘PN40024’ and ‘Sugraone’ genome assemblies. Table S6. WRKY03‐binding events on PME genes detected by DAP‐seq analysis. Table S7. PME10 DAP‐seq qPCR conditions. Table S8. List of primers used throughout the study. Methods S1. Molecular analysis and acclimation procedures for PME10 OE and KO lines. Methods S2. Detailed procedures for Bc artificial inoculation assays. Metho [file PBI-23-4981-s002.zip › pbi70279-sup-0002-FigureS1.png]

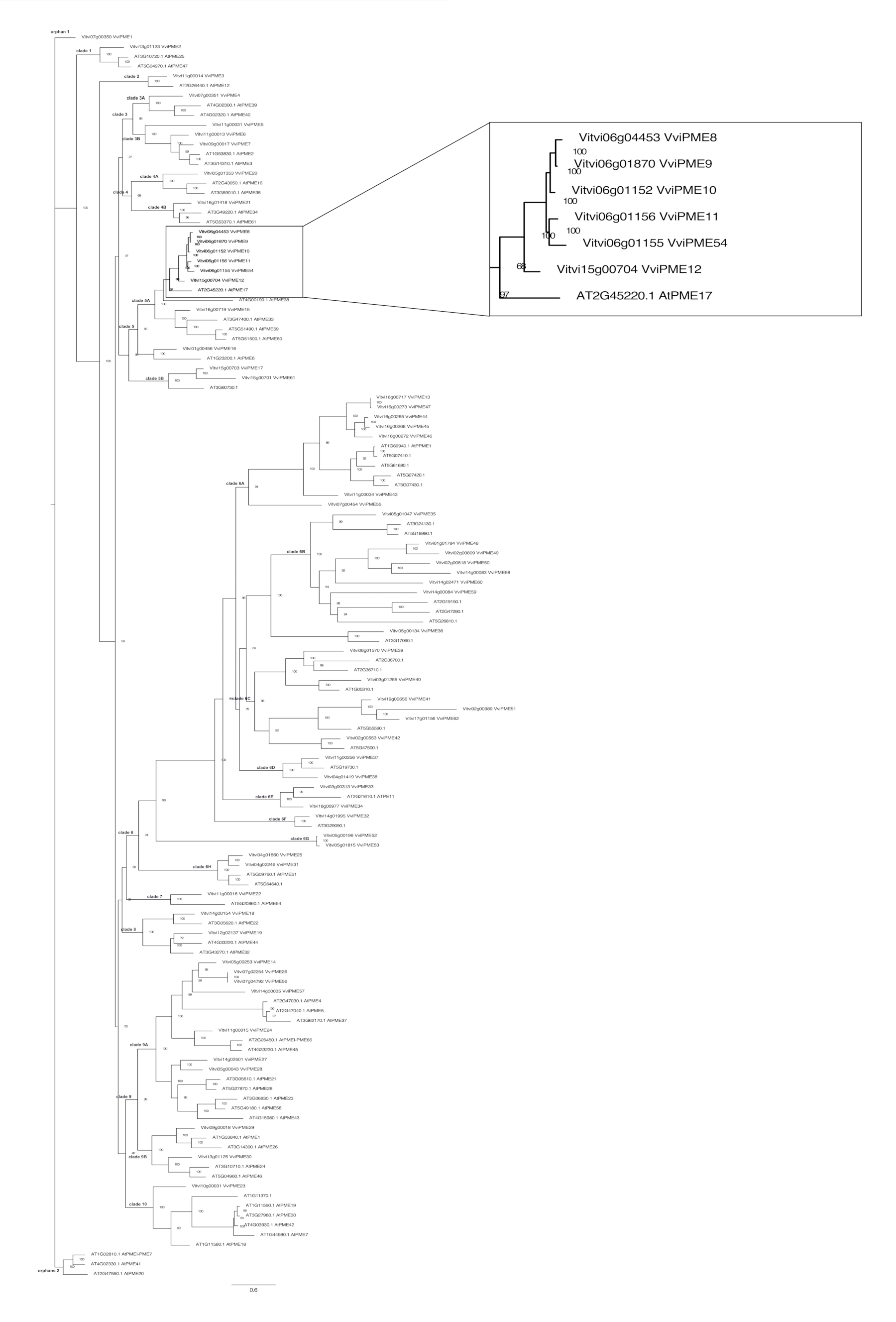

Supplement: Supplementary file 2 — Figure S1. Monosaccharide compositions of cell wall extracts from flowers and berry skins of different grapevine genotypes. Figure S2. Phylogenetic tree of Arabidopsis thaliana and Vitis vinifera Pectin Methyl Esterase (PME) genes. Figure S3. Expression profiles of PME family genes across various grapevine organs and tissues at different developmental stages. Figure S4. Summary of RNA‐seq results comparing Bc‐infected and control flowers of ‘Souvigner Gris’ (SG) and ‘Teroldego’ (TE) at 24 h post‐inoculation. Figure S5. Summary of on‐target analysis of the PME10 knockout (KO) lines. Figure S6. Summary of the off‐target analysis of the PME10 KO lines. Figure S7. Phenotypic characterisation of PME10 KO lines compared with control plants. Figure S8. Phenotypic characterisation of PME10 overexpressing (OE) lines compared with control plants. Figure S9. WRKY03 DAP‐seq and DAP‐qPCR analyses of the WRKY03‐PME10 interaction. Figure S10. Melting curve analysis during qPCR assays using primers for PME10, PME11, and PME12. Table S1. Complete list of the 62 PME genes identified in the V. vinifera PN40024 reference genome. Table S2. Summary of the Illumina read processing and mapping to the concatenated V. vinifera PN40024 12X.v2 and B. cinerea DW1 genome assemblies. Table S3. Differentially expressed genes in ‘Souvigner Gris’ and V. vinifera ‘Teroldego’ flowers at 24 h post‐inoculation with B. cinerea. Table S4. Metadata of publicly available RNA‐seq experiments on B. cinerea ‐grapevine berry interactions, included in the Botrytis Stress Atlas Explorer. Table S5. Predicted PME10 off‐target regions in V. vinifera ‘PN40024’ and ‘Sugraone’ genome assemblies. Table S6. WRKY03‐binding events on PME genes detected by DAP‐seq analysis. Table S7. PME10 DAP‐seq qPCR conditions. Table S8. List of primers used throughout the study. Methods S1. Molecular analysis and acclimation procedures for PME10 OE and KO lines. Methods S2. Detailed procedures for Bc artificial inoculation assays. Metho [file PBI-23-4981-s002.zip › pbi70279-sup-0003-FigureS2.png]

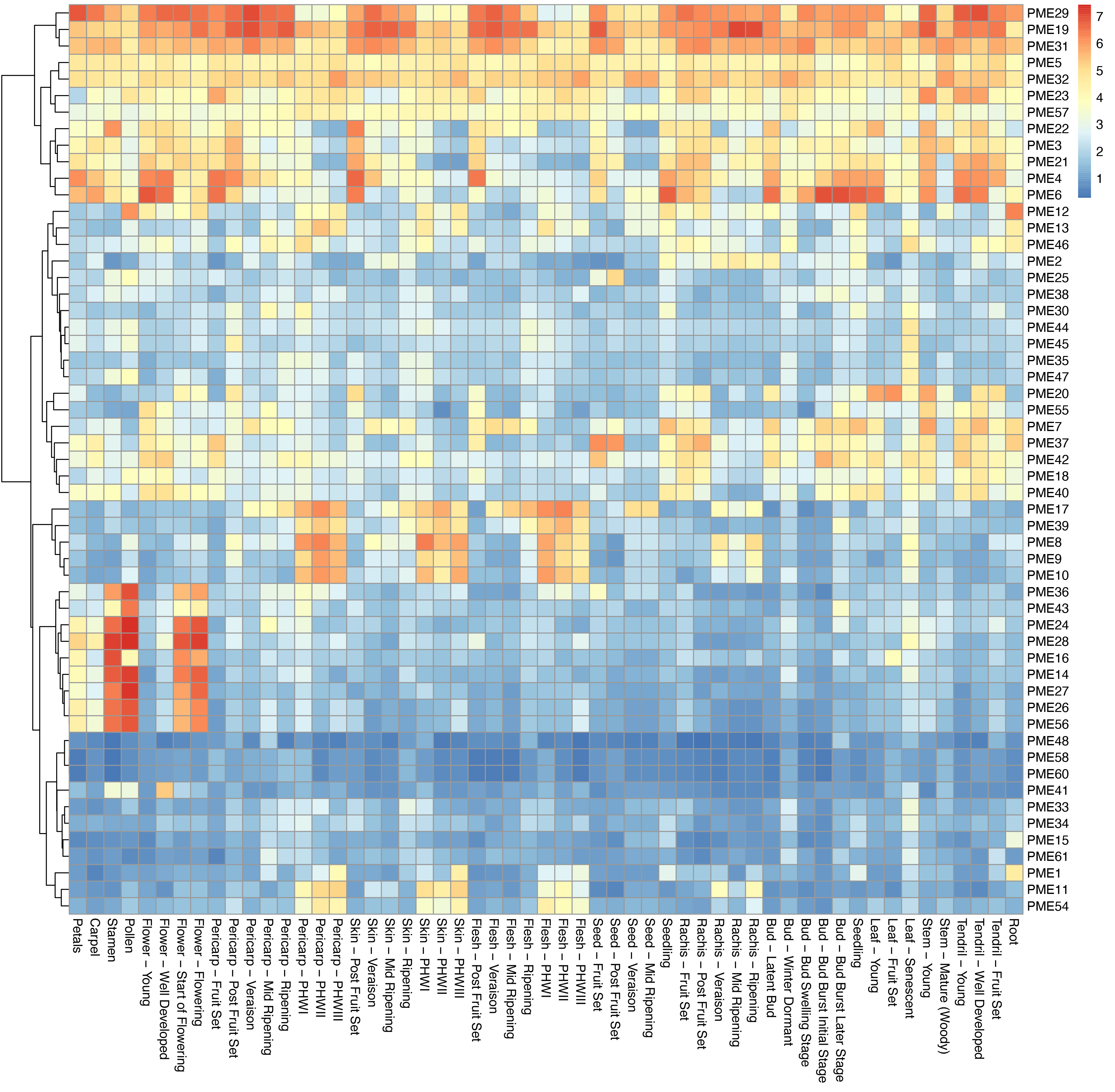

Supplement: Supplementary file 2 — Figure S1. Monosaccharide compositions of cell wall extracts from flowers and berry skins of different grapevine genotypes. Figure S2. Phylogenetic tree of Arabidopsis thaliana and Vitis vinifera Pectin Methyl Esterase (PME) genes. Figure S3. Expression profiles of PME family genes across various grapevine organs and tissues at different developmental stages. Figure S4. Summary of RNA‐seq results comparing Bc‐infected and control flowers of ‘Souvigner Gris’ (SG) and ‘Teroldego’ (TE) at 24 h post‐inoculation. Figure S5. Summary of on‐target analysis of the PME10 knockout (KO) lines. Figure S6. Summary of the off‐target analysis of the PME10 KO lines. Figure S7. Phenotypic characterisation of PME10 KO lines compared with control plants. Figure S8. Phenotypic characterisation of PME10 overexpressing (OE) lines compared with control plants. Figure S9. WRKY03 DAP‐seq and DAP‐qPCR analyses of the WRKY03‐PME10 interaction. Figure S10. Melting curve analysis during qPCR assays using primers for PME10, PME11, and PME12. Table S1. Complete list of the 62 PME genes identified in the V. vinifera PN40024 reference genome. Table S2. Summary of the Illumina read processing and mapping to the concatenated V. vinifera PN40024 12X.v2 and B. cinerea DW1 genome assemblies. Table S3. Differentially expressed genes in ‘Souvigner Gris’ and V. vinifera ‘Teroldego’ flowers at 24 h post‐inoculation with B. cinerea. Table S4. Metadata of publicly available RNA‐seq experiments on B. cinerea ‐grapevine berry interactions, included in the Botrytis Stress Atlas Explorer. Table S5. Predicted PME10 off‐target regions in V. vinifera ‘PN40024’ and ‘Sugraone’ genome assemblies. Table S6. WRKY03‐binding events on PME genes detected by DAP‐seq analysis. Table S7. PME10 DAP‐seq qPCR conditions. Table S8. List of primers used throughout the study. Methods S1. Molecular analysis and acclimation procedures for PME10 OE and KO lines. Methods S2. Detailed procedures for Bc artificial inoculation assays. Metho [file PBI-23-4981-s002.zip › pbi70279-sup-0004-FigureS3.png]

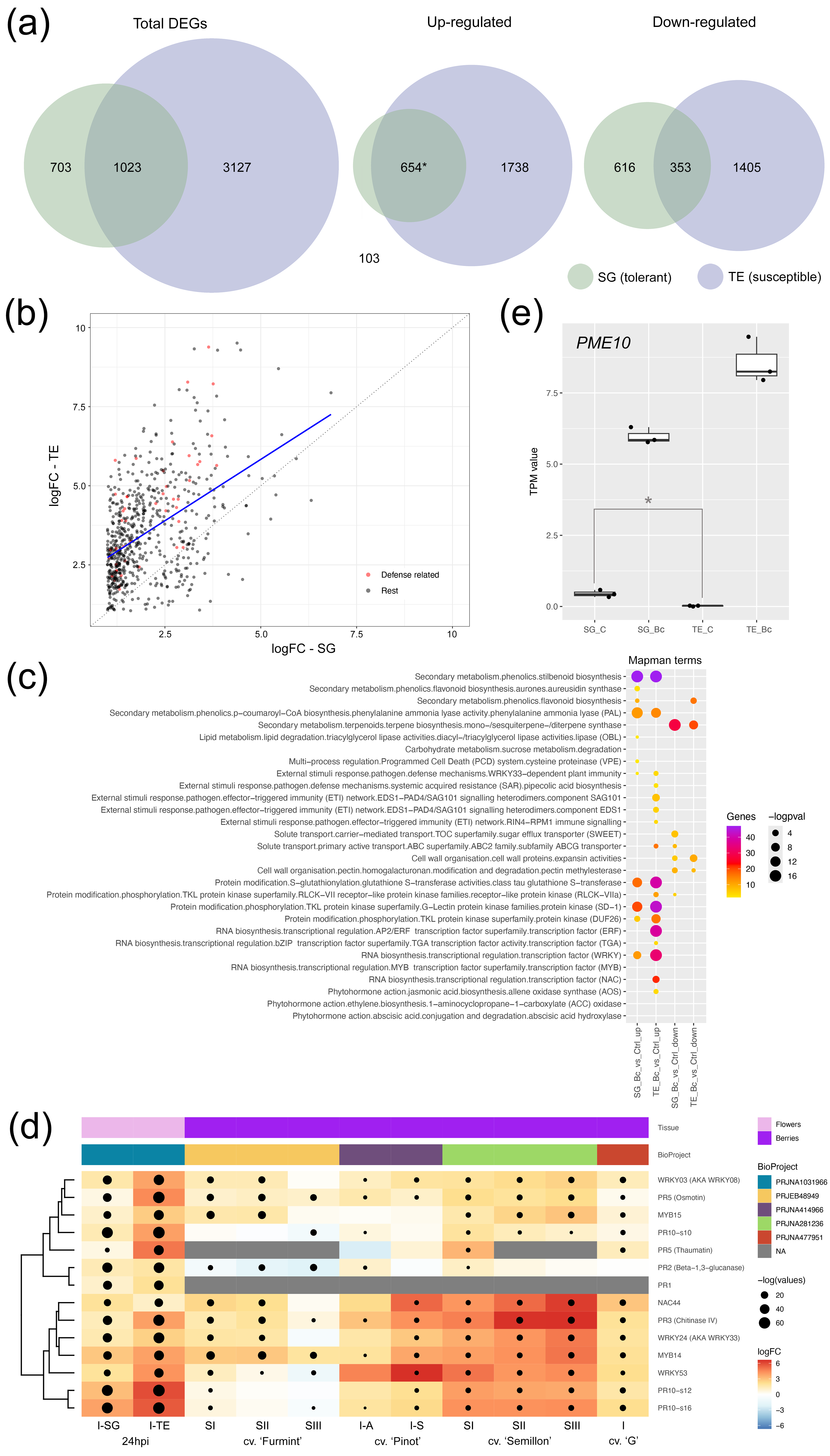

Supplement: Supplementary file 2 — Figure S1. Monosaccharide compositions of cell wall extracts from flowers and berry skins of different grapevine genotypes. Figure S2. Phylogenetic tree of Arabidopsis thaliana and Vitis vinifera Pectin Methyl Esterase (PME) genes. Figure S3. Expression profiles of PME family genes across various grapevine organs and tissues at different developmental stages. Figure S4. Summary of RNA‐seq results comparing Bc‐infected and control flowers of ‘Souvigner Gris’ (SG) and ‘Teroldego’ (TE) at 24 h post‐inoculation. Figure S5. Summary of on‐target analysis of the PME10 knockout (KO) lines. Figure S6. Summary of the off‐target analysis of the PME10 KO lines. Figure S7. Phenotypic characterisation of PME10 KO lines compared with control plants. Figure S8. Phenotypic characterisation of PME10 overexpressing (OE) lines compared with control plants. Figure S9. WRKY03 DAP‐seq and DAP‐qPCR analyses of the WRKY03‐PME10 interaction. Figure S10. Melting curve analysis during qPCR assays using primers for PME10, PME11, and PME12. Table S1. Complete list of the 62 PME genes identified in the V. vinifera PN40024 reference genome. Table S2. Summary of the Illumina read processing and mapping to the concatenated V. vinifera PN40024 12X.v2 and B. cinerea DW1 genome assemblies. Table S3. Differentially expressed genes in ‘Souvigner Gris’ and V. vinifera ‘Teroldego’ flowers at 24 h post‐inoculation with B. cinerea. Table S4. Metadata of publicly available RNA‐seq experiments on B. cinerea ‐grapevine berry interactions, included in the Botrytis Stress Atlas Explorer. Table S5. Predicted PME10 off‐target regions in V. vinifera ‘PN40024’ and ‘Sugraone’ genome assemblies. Table S6. WRKY03‐binding events on PME genes detected by DAP‐seq analysis. Table S7. PME10 DAP‐seq qPCR conditions. Table S8. List of primers used throughout the study. Methods S1. Molecular analysis and acclimation procedures for PME10 OE and KO lines. Methods S2. Detailed procedures for Bc artificial inoculation assays. Metho [file PBI-23-4981-s002.zip › pbi70279-sup-0005-FigureS4.png]

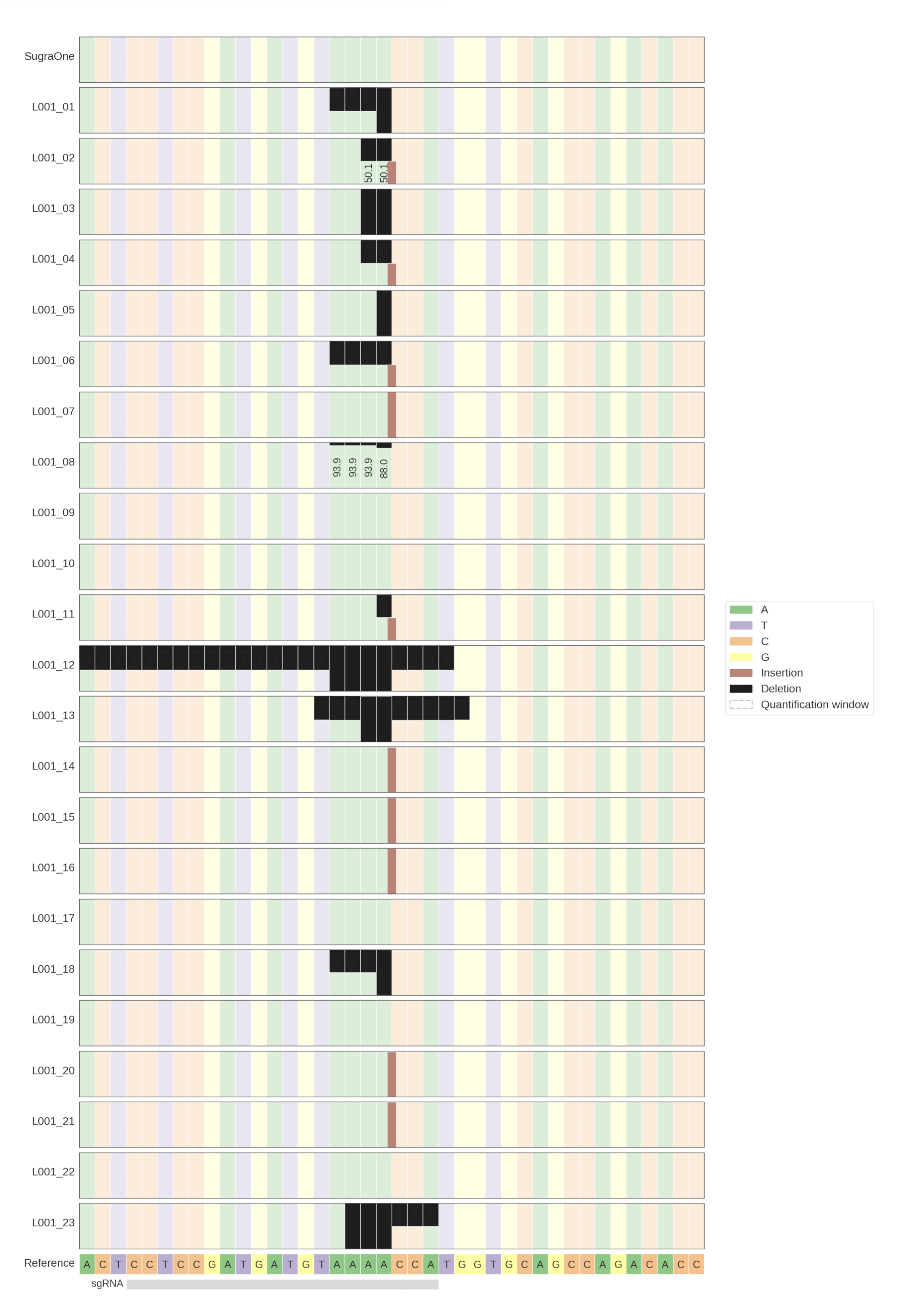

Supplement: Supplementary file 2 — Figure S1. Monosaccharide compositions of cell wall extracts from flowers and berry skins of different grapevine genotypes. Figure S2. Phylogenetic tree of Arabidopsis thaliana and Vitis vinifera Pectin Methyl Esterase (PME) genes. Figure S3. Expression profiles of PME family genes across various grapevine organs and tissues at different developmental stages. Figure S4. Summary of RNA‐seq results comparing Bc‐infected and control flowers of ‘Souvigner Gris’ (SG) and ‘Teroldego’ (TE) at 24 h post‐inoculation. Figure S5. Summary of on‐target analysis of the PME10 knockout (KO) lines. Figure S6. Summary of the off‐target analysis of the PME10 KO lines. Figure S7. Phenotypic characterisation of PME10 KO lines compared with control plants. Figure S8. Phenotypic characterisation of PME10 overexpressing (OE) lines compared with control plants. Figure S9. WRKY03 DAP‐seq and DAP‐qPCR analyses of the WRKY03‐PME10 interaction. Figure S10. Melting curve analysis during qPCR assays using primers for PME10, PME11, and PME12. Table S1. Complete list of the 62 PME genes identified in the V. vinifera PN40024 reference genome. Table S2. Summary of the Illumina read processing and mapping to the concatenated V. vinifera PN40024 12X.v2 and B. cinerea DW1 genome assemblies. Table S3. Differentially expressed genes in ‘Souvigner Gris’ and V. vinifera ‘Teroldego’ flowers at 24 h post‐inoculation with B. cinerea. Table S4. Metadata of publicly available RNA‐seq experiments on B. cinerea ‐grapevine berry interactions, included in the Botrytis Stress Atlas Explorer. Table S5. Predicted PME10 off‐target regions in V. vinifera ‘PN40024’ and ‘Sugraone’ genome assemblies. Table S6. WRKY03‐binding events on PME genes detected by DAP‐seq analysis. Table S7. PME10 DAP‐seq qPCR conditions. Table S8. List of primers used throughout the study. Methods S1. Molecular analysis and acclimation procedures for PME10 OE and KO lines. Methods S2. Detailed procedures for Bc artificial inoculation assays. Metho [file PBI-23-4981-s002.zip › pbi70279-sup-0006-FigureS5.png]

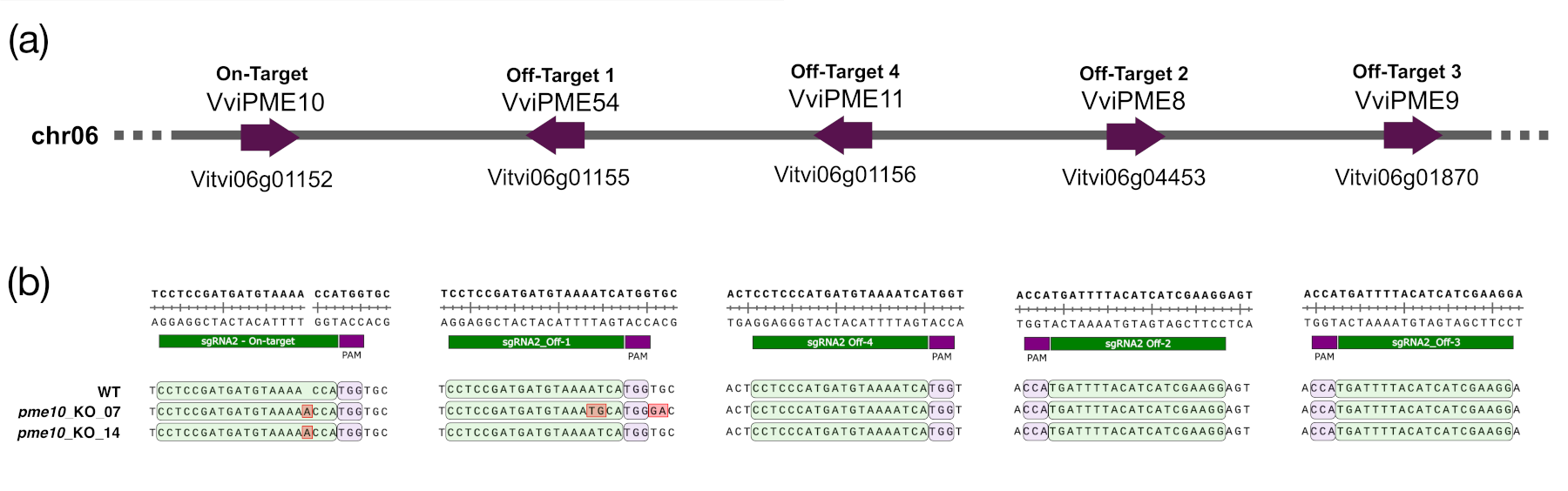

Supplement: Supplementary file 2 — Figure S1. Monosaccharide compositions of cell wall extracts from flowers and berry skins of different grapevine genotypes. Figure S2. Phylogenetic tree of Arabidopsis thaliana and Vitis vinifera Pectin Methyl Esterase (PME) genes. Figure S3. Expression profiles of PME family genes across various grapevine organs and tissues at different developmental stages. Figure S4. Summary of RNA‐seq results comparing Bc‐infected and control flowers of ‘Souvigner Gris’ (SG) and ‘Teroldego’ (TE) at 24 h post‐inoculation. Figure S5. Summary of on‐target analysis of the PME10 knockout (KO) lines. Figure S6. Summary of the off‐target analysis of the PME10 KO lines. Figure S7. Phenotypic characterisation of PME10 KO lines compared with control plants. Figure S8. Phenotypic characterisation of PME10 overexpressing (OE) lines compared with control plants. Figure S9. WRKY03 DAP‐seq and DAP‐qPCR analyses of the WRKY03‐PME10 interaction. Figure S10. Melting curve analysis during qPCR assays using primers for PME10, PME11, and PME12. Table S1. Complete list of the 62 PME genes identified in the V. vinifera PN40024 reference genome. Table S2. Summary of the Illumina read processing and mapping to the concatenated V. vinifera PN40024 12X.v2 and B. cinerea DW1 genome assemblies. Table S3. Differentially expressed genes in ‘Souvigner Gris’ and V. vinifera ‘Teroldego’ flowers at 24 h post‐inoculation with B. cinerea. Table S4. Metadata of publicly available RNA‐seq experiments on B. cinerea ‐grapevine berry interactions, included in the Botrytis Stress Atlas Explorer. Table S5. Predicted PME10 off‐target regions in V. vinifera ‘PN40024’ and ‘Sugraone’ genome assemblies. Table S6. WRKY03‐binding events on PME genes detected by DAP‐seq analysis. Table S7. PME10 DAP‐seq qPCR conditions. Table S8. List of primers used throughout the study. Methods S1. Molecular analysis and acclimation procedures for PME10 OE and KO lines. Methods S2. Detailed procedures for Bc artificial inoculation assays. Metho [file PBI-23-4981-s002.zip › pbi70279-sup-0007-FigureS6.png]

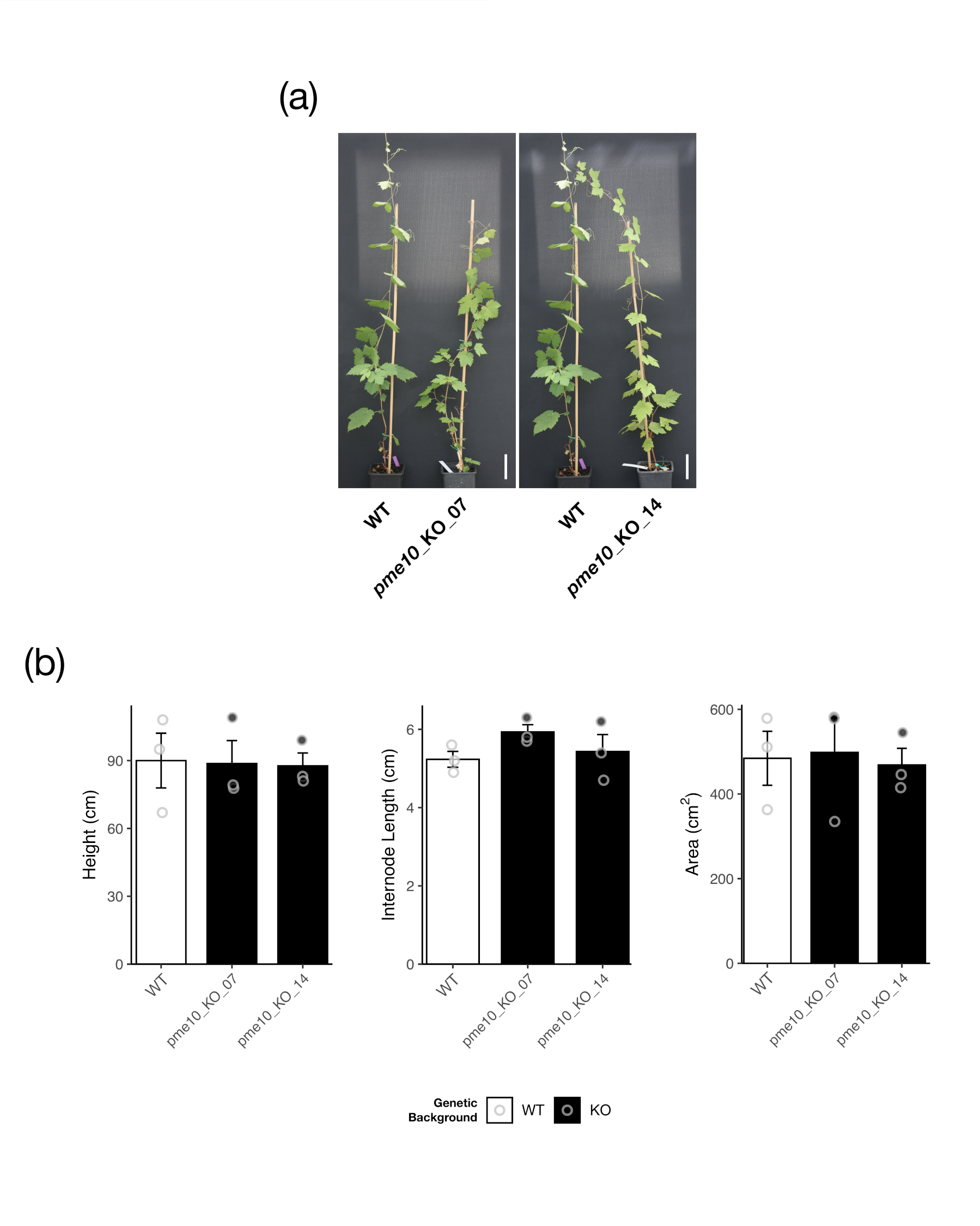

Supplement: Supplementary file 2 — Figure S1. Monosaccharide compositions of cell wall extracts from flowers and berry skins of different grapevine genotypes. Figure S2. Phylogenetic tree of Arabidopsis thaliana and Vitis vinifera Pectin Methyl Esterase (PME) genes. Figure S3. Expression profiles of PME family genes across various grapevine organs and tissues at different developmental stages. Figure S4. Summary of RNA‐seq results comparing Bc‐infected and control flowers of ‘Souvigner Gris’ (SG) and ‘Teroldego’ (TE) at 24 h post‐inoculation. Figure S5. Summary of on‐target analysis of the PME10 knockout (KO) lines. Figure S6. Summary of the off‐target analysis of the PME10 KO lines. Figure S7. Phenotypic characterisation of PME10 KO lines compared with control plants. Figure S8. Phenotypic characterisation of PME10 overexpressing (OE) lines compared with control plants. Figure S9. WRKY03 DAP‐seq and DAP‐qPCR analyses of the WRKY03‐PME10 interaction. Figure S10. Melting curve analysis during qPCR assays using primers for PME10, PME11, and PME12. Table S1. Complete list of the 62 PME genes identified in the V. vinifera PN40024 reference genome. Table S2. Summary of the Illumina read processing and mapping to the concatenated V. vinifera PN40024 12X.v2 and B. cinerea DW1 genome assemblies. Table S3. Differentially expressed genes in ‘Souvigner Gris’ and V. vinifera ‘Teroldego’ flowers at 24 h post‐inoculation with B. cinerea. Table S4. Metadata of publicly available RNA‐seq experiments on B. cinerea ‐grapevine berry interactions, included in the Botrytis Stress Atlas Explorer. Table S5. Predicted PME10 off‐target regions in V. vinifera ‘PN40024’ and ‘Sugraone’ genome assemblies. Table S6. WRKY03‐binding events on PME genes detected by DAP‐seq analysis. Table S7. PME10 DAP‐seq qPCR conditions. Table S8. List of primers used throughout the study. Methods S1. Molecular analysis and acclimation procedures for PME10 OE and KO lines. Methods S2. Detailed procedures for Bc artificial inoculation assays. Metho [file PBI-23-4981-s002.zip › pbi70279-sup-0008-FigureS7.png]

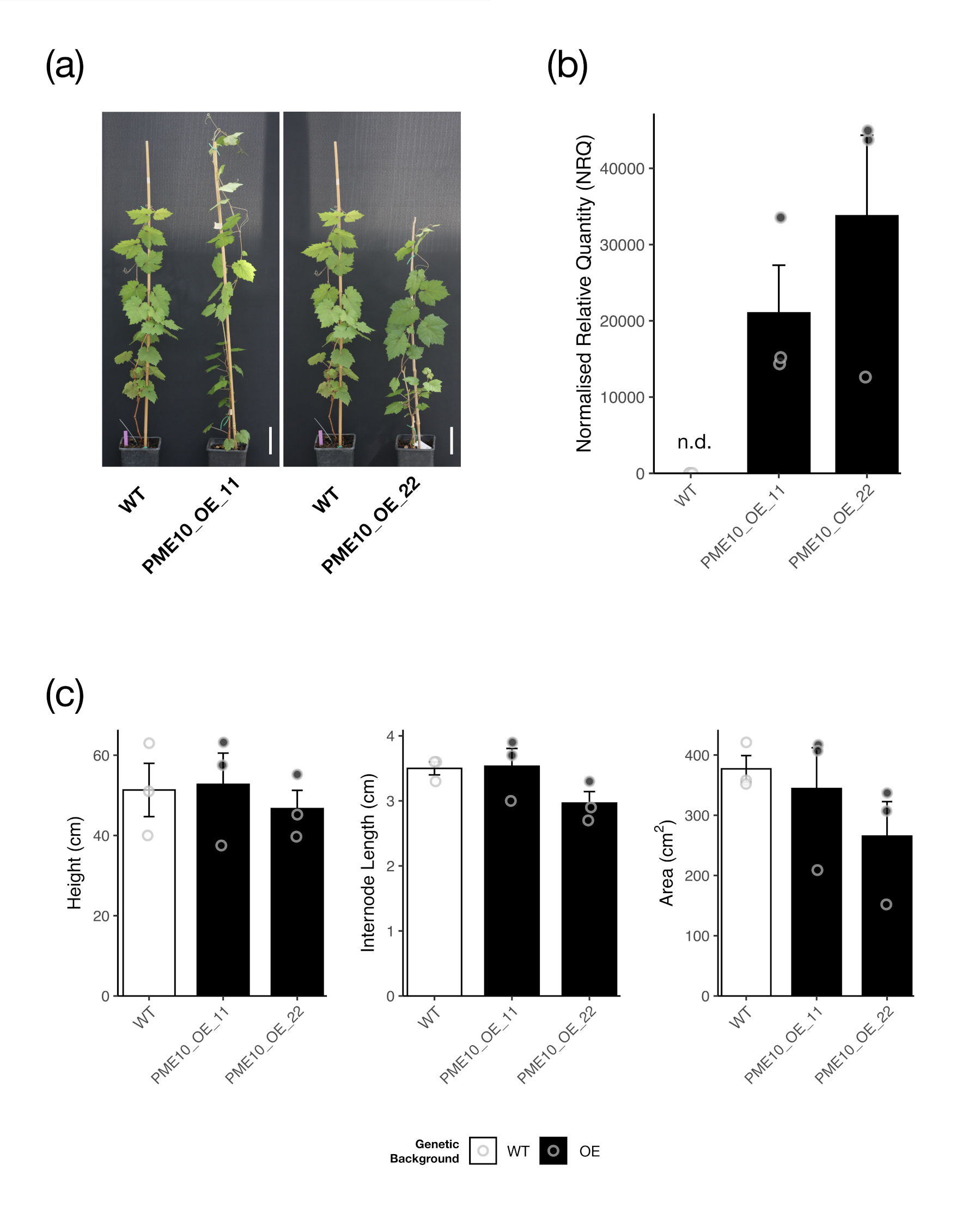

Supplement: Supplementary file 2 — Figure S1. Monosaccharide compositions of cell wall extracts from flowers and berry skins of different grapevine genotypes. Figure S2. Phylogenetic tree of Arabidopsis thaliana and Vitis vinifera Pectin Methyl Esterase (PME) genes. Figure S3. Expression profiles of PME family genes across various grapevine organs and tissues at different developmental stages. Figure S4. Summary of RNA‐seq results comparing Bc‐infected and control flowers of ‘Souvigner Gris’ (SG) and ‘Teroldego’ (TE) at 24 h post‐inoculation. Figure S5. Summary of on‐target analysis of the PME10 knockout (KO) lines. Figure S6. Summary of the off‐target analysis of the PME10 KO lines. Figure S7. Phenotypic characterisation of PME10 KO lines compared with control plants. Figure S8. Phenotypic characterisation of PME10 overexpressing (OE) lines compared with control plants. Figure S9. WRKY03 DAP‐seq and DAP‐qPCR analyses of the WRKY03‐PME10 interaction. Figure S10. Melting curve analysis during qPCR assays using primers for PME10, PME11, and PME12. Table S1. Complete list of the 62 PME genes identified in the V. vinifera PN40024 reference genome. Table S2. Summary of the Illumina read processing and mapping to the concatenated V. vinifera PN40024 12X.v2 and B. cinerea DW1 genome assemblies. Table S3. Differentially expressed genes in ‘Souvigner Gris’ and V. vinifera ‘Teroldego’ flowers at 24 h post‐inoculation with B. cinerea. Table S4. Metadata of publicly available RNA‐seq experiments on B. cinerea ‐grapevine berry interactions, included in the Botrytis Stress Atlas Explorer. Table S5. Predicted PME10 off‐target regions in V. vinifera ‘PN40024’ and ‘Sugraone’ genome assemblies. Table S6. WRKY03‐binding events on PME genes detected by DAP‐seq analysis. Table S7. PME10 DAP‐seq qPCR conditions. Table S8. List of primers used throughout the study. Methods S1. Molecular analysis and acclimation procedures for PME10 OE and KO lines. Methods S2. Detailed procedures for Bc artificial inoculation assays. Metho [file PBI-23-4981-s002.zip › pbi70279-sup-0009-FigureS8.png]

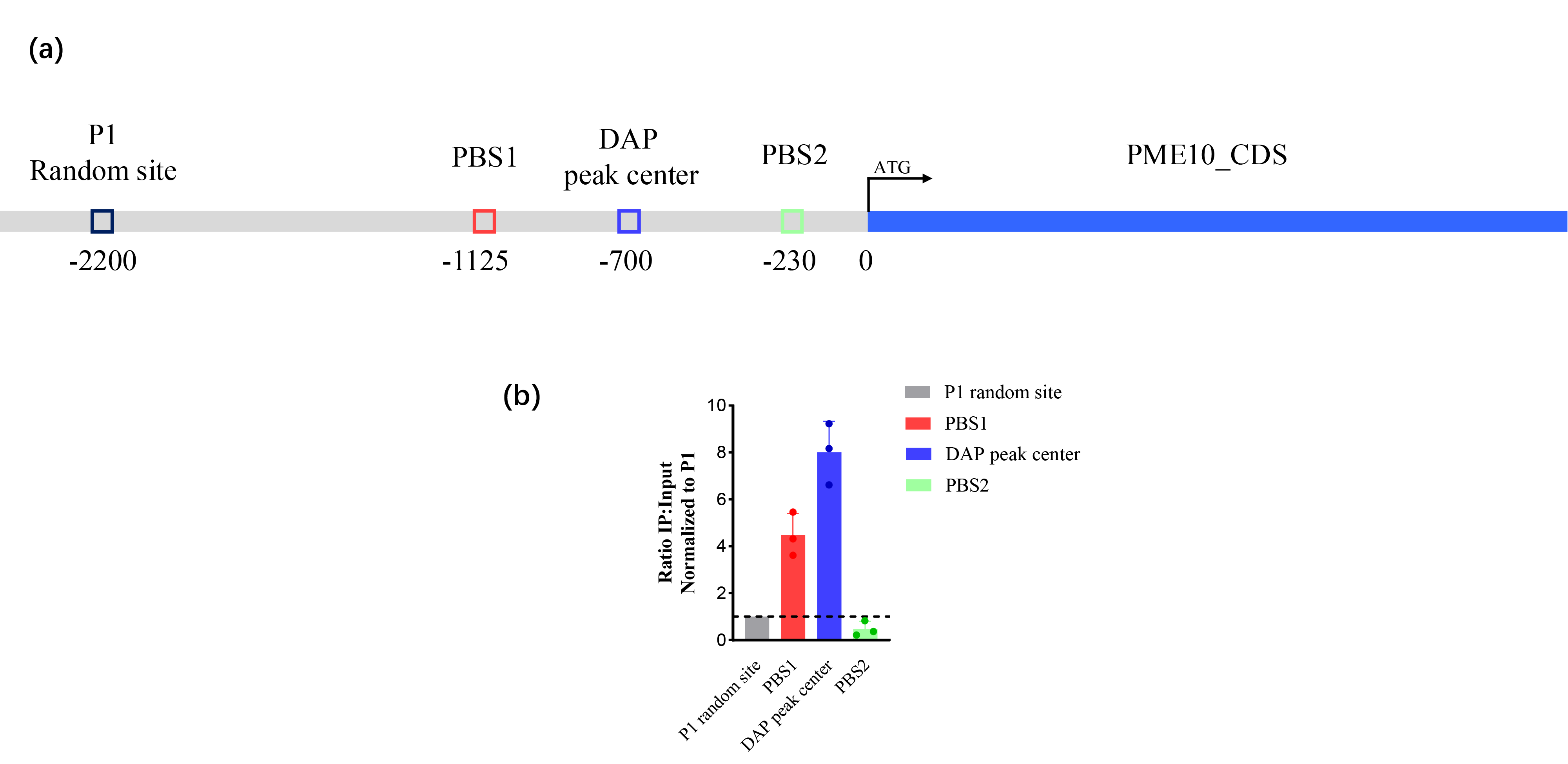

Supplement: Supplementary file 2 — Figure S1. Monosaccharide compositions of cell wall extracts from flowers and berry skins of different grapevine genotypes. Figure S2. Phylogenetic tree of Arabidopsis thaliana and Vitis vinifera Pectin Methyl Esterase (PME) genes. Figure S3. Expression profiles of PME family genes across various grapevine organs and tissues at different developmental stages. Figure S4. Summary of RNA‐seq results comparing Bc‐infected and control flowers of ‘Souvigner Gris’ (SG) and ‘Teroldego’ (TE) at 24 h post‐inoculation. Figure S5. Summary of on‐target analysis of the PME10 knockout (KO) lines. Figure S6. Summary of the off‐target analysis of the PME10 KO lines. Figure S7. Phenotypic characterisation of PME10 KO lines compared with control plants. Figure S8. Phenotypic characterisation of PME10 overexpressing (OE) lines compared with control plants. Figure S9. WRKY03 DAP‐seq and DAP‐qPCR analyses of the WRKY03‐PME10 interaction. Figure S10. Melting curve analysis during qPCR assays using primers for PME10, PME11, and PME12. Table S1. Complete list of the 62 PME genes identified in the V. vinifera PN40024 reference genome. Table S2. Summary of the Illumina read processing and mapping to the concatenated V. vinifera PN40024 12X.v2 and B. cinerea DW1 genome assemblies. Table S3. Differentially expressed genes in ‘Souvigner Gris’ and V. vinifera ‘Teroldego’ flowers at 24 h post‐inoculation with B. cinerea. Table S4. Metadata of publicly available RNA‐seq experiments on B. cinerea ‐grapevine berry interactions, included in the Botrytis Stress Atlas Explorer. Table S5. Predicted PME10 off‐target regions in V. vinifera ‘PN40024’ and ‘Sugraone’ genome assemblies. Table S6. WRKY03‐binding events on PME genes detected by DAP‐seq analysis. Table S7. PME10 DAP‐seq qPCR conditions. Table S8. List of primers used throughout the study. Methods S1. Molecular analysis and acclimation procedures for PME10 OE and KO lines. Methods S2. Detailed procedures for Bc artificial inoculation assays. Metho [file PBI-23-4981-s002.zip › pbi70279-sup-0010-FigureS9.png]

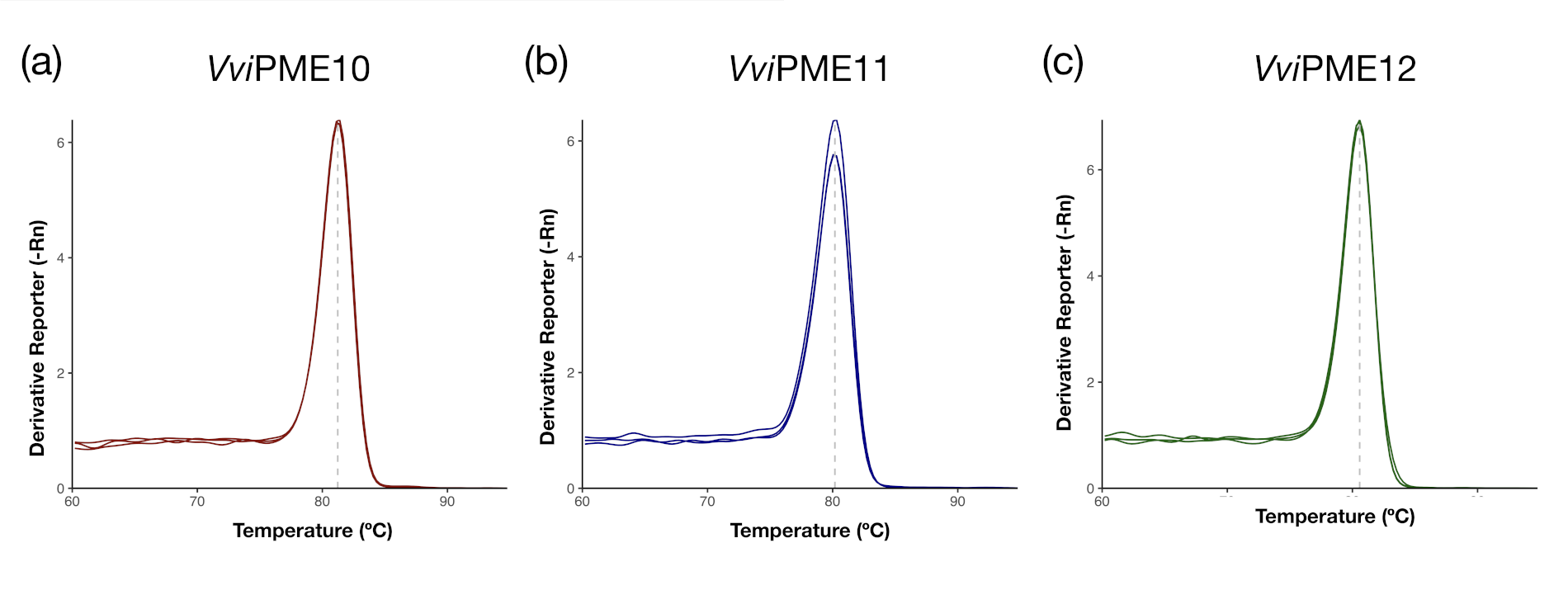

Supplement: Supplementary file 2 — Figure S1. Monosaccharide compositions of cell wall extracts from flowers and berry skins of different grapevine genotypes. Figure S2. Phylogenetic tree of Arabidopsis thaliana and Vitis vinifera Pectin Methyl Esterase (PME) genes. Figure S3. Expression profiles of PME family genes across various grapevine organs and tissues at different developmental stages. Figure S4. Summary of RNA‐seq results comparing Bc‐infected and control flowers of ‘Souvigner Gris’ (SG) and ‘Teroldego’ (TE) at 24 h post‐inoculation. Figure S5. Summary of on‐target analysis of the PME10 knockout (KO) lines. Figure S6. Summary of the off‐target analysis of the PME10 KO lines. Figure S7. Phenotypic characterisation of PME10 KO lines compared with control plants. Figure S8. Phenotypic characterisation of PME10 overexpressing (OE) lines compared with control plants. Figure S9. WRKY03 DAP‐seq and DAP‐qPCR analyses of the WRKY03‐PME10 interaction. Figure S10. Melting curve analysis during qPCR assays using primers for PME10, PME11, and PME12. Table S1. Complete list of the 62 PME genes identified in the V. vinifera PN40024 reference genome. Table S2. Summary of the Illumina read processing and mapping to the concatenated V. vinifera PN40024 12X.v2 and B. cinerea DW1 genome assemblies. Table S3. Differentially expressed genes in ‘Souvigner Gris’ and V. vinifera ‘Teroldego’ flowers at 24 h post‐inoculation with B. cinerea. Table S4. Metadata of publicly available RNA‐seq experiments on B. cinerea ‐grapevine berry interactions, included in the Botrytis Stress Atlas Explorer. Table S5. Predicted PME10 off‐target regions in V. vinifera ‘PN40024’ and ‘Sugraone’ genome assemblies. Table S6. WRKY03‐binding events on PME genes detected by DAP‐seq analysis. Table S7. PME10 DAP‐seq qPCR conditions. Table S8. List of primers used throughout the study. Methods S1. Molecular analysis and acclimation procedures for PME10 OE and KO lines. Methods S2. Detailed procedures for Bc artificial inoculation assays. Metho [file PBI-23-4981-s002.zip › pbi70279-sup-0011-FigureS10.png]
